# SimTac: A Physics-Based Simulator for Vision-Based Tactile Sensing with Biomorphic Structures

**DOI:** 10.34133/cbsystems.0510

**Published:** 2026-02-24

**Authors:** Xuyang Zhang, Jiaqi Jiang, Zhuo Chen, Yongqiang Zhao, Tianqi Yang, Daniel Fernandes Gomes, Jianan Wang, Shan Luo

**Affiliations:** ^1^ Department of Engineering, King’s College London, London WC2R 2LS, UK.; ^2^ School of Aerospace Engineering, Beijing Institute of Technology, Beijing 100081, China.; ^3^ Department of Computer Science, University College London, London WC1V 6LJ, UK.; ^4^ Institute for Systems and Computer Engineering, Technology and Science,Porto 4200-465, Portugal.

## Abstract

Tactile sensing in biological organisms is deeply intertwined with morphological form, such as human fingers, cat paws, and elephant trunks, which enables rich and adaptive interactions through a variety of geometrically complex structures. In contrast, vision-based tactile sensors in robotics have been limited to simple planar geometries, with biomorphic designs remaining underexplored. To address this gap, we present SimTac, a physics-based simulation framework for the design and validation of biomorphic tactile sensors. SimTac consists of particle-based deformation modeling, light-field rendering for photorealistic tactile image generation, and a neural network for predicting mechanical responses, enabling accurate and efficient simulation across a wide range of geometries and materials. We demonstrate the versatility of SimTac by designing and validating physical sensor prototypes inspired by biological tactile structures and further demonstrate its effectiveness across multiple Sim2Real tactile tasks, including object classification, slip detection, and contact safety assessment. Our framework bridges the gap between bioinspired design and practical realization, expanding the design space of tactile sensors and paving the way for tactile sensing systems that integrate morphology and sensing to enable robust interaction in unstructured environments.

## Introduction

Biological organisms have evolved a wide variety of tactile sensing structures, ranging from the dexterous fingers of humans and the retractable claws of cats to the flexible tentacles of octopus and the prehensile trunks of elephants [1,2]. The morphological diversity of these structures is closely linked to their functional adaptability: the soft, tubular shape and sucker structures of the octopus tentacle allow it to conform to complex surfaces, while the elephant’s trunk tip, with 2 finger-like protrusions, enables it to pinch and grasp a variety of objects. The tactile sensing capabilities of these structures further enable the perception of object properties, such as shape, texture, and stiffness, while also facilitating adaptive interactions with the environment. For instance, tactile sensing allows an organism to detect and respond to external forces, such as adjusting grip to prevent an object from slipping or rapidly contracting in response to harmful stimuli for safety [3]. The synergy between morphological diversity and tactile sensing in biological systems serves as a powerful source of inspiration for the design of tactile sensors.

Tactile sensors, such as resistive [4], capacitive [5], and photoresistive [6] types, are widely used in robotics for detecting contact forces and object deformations. However, conventional tactile sensors often struggle with shape adaptability and the precise capture of intricate contact details. In contrast, vision-based tactile sensors have recently emerged as a highly promising technology for achieving high-resolution tactile perception [7–19]. These sensors typically consist of a camera positioned underneath a soft elastomer layer, capturing visual data of the elastomer’s deformation upon contact with an object. By analyzing these deformations in captured images, a wealth of physical information can be extracted, including surface textures [20], object pose [21,22], shape [23], slip detection [24], and contact forces [25]. These rich data enable enhanced robotic dexterity in unstructured environments. However, existing sensors are typically limited to simple geometric shapes, such as cubic [7] and hemispherical forms [12]. To improve adaptability for interacting with complex surfaces, recent research has focused on developing more advanced sensor morphologies, such as finger-like structures [10,14]. These innovations not only expand the sensing area but also enhance the sensor’s ability to conform to diverse object geometries, thereby enhancing robotic perception.

Designing vision-based tactile sensors with biomorphic forms through trial-and-error and iterative hardware prototyping is challenging [10,14]. Unlike simple geometric shapes, natural and organic morphologies introduce more complex skin geometries, making the deformation upon contact with objects difficult to model and estimate. Integrating key components such as cameras and light-emitting diodes (LEDs) within these complex geometries further complicates the design process, as it requires establishing well-controlled light paths and ensuring optimal imaging of the contact regions [9,10]. In addition, learning-based methods for robotic perception and control typically require large-scale data collection [20,26]. In this process, the high cost and time-consuming data collection, along with the physical wear and tear of sensors, present a major bottleneck.

**Fig. 1.**
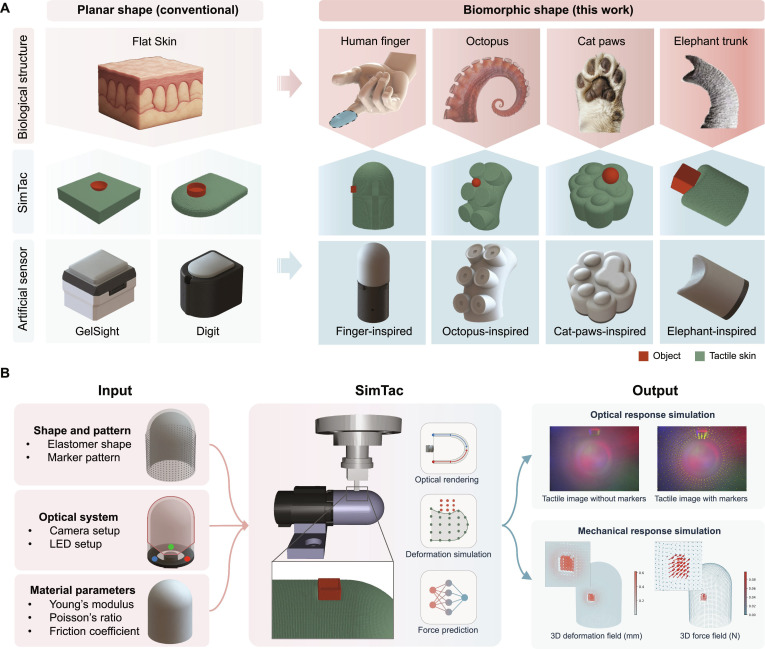
Bridging biological and artificial tactile sensing via SimTac: a simulator for modeling biomorphic-vision-based tactile sensors. (A) Compared with conventional flat-shaped tactile sensors, biomorphically inspired tactile sensors exhibit enhanced geometric complexity and an extended sensing range, which imposes increased challenges for simulation modeling. (B) The simulation framework of SimTac. The input includes the sensor shape, marker pattern, optical system definition, and material properties. The output consists of optical responses and mechanical responses.

Simulation offers an efficient and repeatable approach to replicating physical interactions in a virtual environment [27,28], facilitating accelerated prototyping, systematic testing, and rapid data collection for vision-based tactile sensors. Such a simulator typically involves 3 key components: deformation simulation to model sensor skin behavior, optical simulation to render tactile images, and force simulation to estimate contact forces. For deformation simulation, depth-based methods [27–32] approximate deformation efficiently but ignore material properties. Physics-based approaches, such as the material point method (MPM)-based approaches [33–38], model material behavior more accurately, but current works are limited to flat sensor geometries. Finite element method (FEM)-based approaches improve accuracy [39–44], yet their computational cost scales substantially with mesh density, posing challenges for real-time interactive applications. For optical simulation, data-driven methods [29,42,43,45–47] learn the color distribution of real tactile images for rendering but struggle with generalization across different sensors, while physics-based rendering, e.g., Phong’s model [27,33], struggles with complex-shaped membranes. Path-tracing-based approaches [34,35,41,48,49] offer realism, although typically at a high computational cost. For force simulation, penalty-based methods [50] are efficient but less accurate than FEM-based approaches [43,50], whose efficiency also degrades greatly with complex shapes. Overall, most existing tactile simulators are limited to flat sensor shapes, while the simulation of biomorphic sensor structures remains largely unexplored, as shown in Fig. 1A.

In this work, we present SimTac, a physics-based simulator for vision-based tactile sensors with biomorphic geometries, capable of generating accurate optical and mechanical responses in real time, as shown in Fig. 1B. SimTac features 3 core components: a particle-based framework to simulate sensor deformation, a light field rendering system for generating high-fidelity tactile images, and a neural network for predicting dense force distributions. The simulator offers exceptional flexibility, supporting a broad range of biomorphic shapes—including human fingers, cat paws, octopus tentacles, and elephant trunks—as well as diverse optical configurations and material properties, from soft elastomers to rigid substrates. SimTac also enables zero-shot sim-to-real transfer across a variety of tactile perception tasks, such as object shape classification, slip detection, and contact safety assessment. By expanding the morphological design space for vision-based tactile sensing, SimTac opens up new possibilities for adaptive robotic systems that tightly couple morphology and sensing to interact more effectively with unstructured environments.

## Materials and Methods

### System overview

The simulator consists of 3 main modules: (a) a particle-based iteration model for the simulation of contact and sensor membrane deformation; (b) a light-field-based lighting model for optical rendering, and (c) a neural network-based model for dense force field mapping. To facilitate understanding of the proposed simulation methodology, the finger-shaped GelTip tactile sensor [10] is used as the simulated target.

### Sensor membrane deformation simulation

**Fig. 2.**
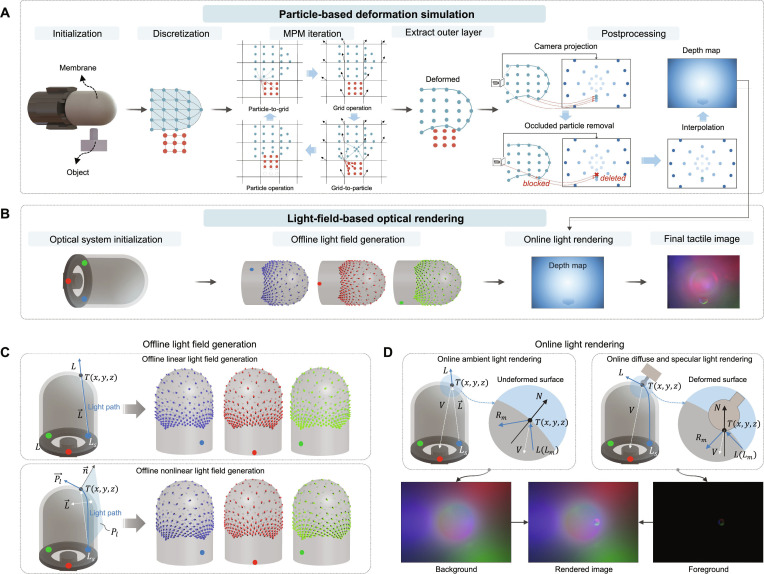
The principle of sensor membrane deformation simulation and optical rendering simulation. Here, we use a finger-shaped GelTip sensor [10] as an example. (A and B) The pipeline of the tactile image simulation. We amplify the contact deformation and sparsify the particle density to facilitate easier understanding; a particle-based iteration method is used for simulating membrane deformation, while optical rendering is achieved using a light-field-based lighting model. (C) Offline light field generation. (D) Real-time online image rendering.

Fig. 2A illustrates the pipeline for simulating the deformation of the sensor membrane. The process begins with the initialization and discretization of both the sensor membrane and the contacting object. A particle-based iterative method is then applied to compute the deformations occurring during the contact. Finally, particles are extracted and served as input for optical rendering and force prediction after postprocessing.

#### Initialization and discretization

We discretize the contacted object into particles using the uniform sampling method provided by Open3D [51]. For the sensor membrane model, we apply the structured mesh algorithm [52] for uniform mesh partitioning, treating mesh nodes as particles, and recording their indices. This discretization strategy enables uniform particleization of sensor membranes with arbitrary geometries while preserving particle indices, thereby facilitating the tracking of particles of interest, such as those located in regions associated with actuators or boundary conditions.

#### Particle-based iteration

After discretizing the sensor membrane and contacting objects, we use the MPM [53], a particle-based iterative approach for simulating sensor deformation. Each particle is assigned properties such as mass, momentum, and stress, which are transferred between the particles and fixed virtual grids during each contact iteration. This process enables the update of particle velocities and displacements, allowing for the calculation of object motion and resulting deformation during interaction (see Note S4.1 for details). Both the sensor membrane and the contacting objects are modeled as elastic bodies, characterized by Young’s modulus E and Poisson’s ratio v. Rigid components are similarly treated as elastic bodies, with rigidity enforced by constraining the velocities of the relevant particles. Active motion is achieved by controlling the velocities of particles within actuator components, while boundary conditions are imposed by constraining the displacements of particles on fixed surfaces. Once the target posture is achieved (e.g., when the indenter or sensor reaches the desired position), the particle-based iteration concludes. We then extract the particles within the outer surface of the deformed membrane for postprocessing.

#### Data postprocessing

The postprocessing pipeline for optical rendering consists of 3 components: occluded particle removal, camera projection, and depth map interpolation. For occluded particle removal, occlusion arises from the overlap between the foreground and background when projecting the deformed 3-dimensional (3D) particle cloud onto the 2D camera coordinate system, which is especially prominent on sensors with complex shapes or large deformations. To remove occluded particles, we utilize a ray casting algorithm [54] on the extracted outer surface: For each particle $P_{i}\left(x_{i},\;y_{i},\;z_{i}\right)$ on the surface, we compute the ray vector ${\vec{L}}_{i}$ from the camera position $C_{c}\left(x_{c},\;y_{c},\;z_{c}\right)$ to this particle and check whether it intersects with any triangle mesh $M_{j}$ within the surface. If ${\vec{L}}_{i}$ intersects any triangle in $M_{j}$ before reaching $P_{i}$, the particle is considered occluded and will be removed.

For the camera projection, the deformed particle cloud *P*, with occluded particles removed, will be projected into a discrete depth map *D* using the following camera projection model, with the camera positioned at $C_{c}\left(x_{c},\;y_{c},\;z_{c}\right)$:

$$u_{i}=\frac{x_{i}}{z_{i}}f_{u}+c_{x},\;v_{i}=\frac{y_{i}}{z_{i}}f_{v}+c_{y},\;d_{i}=\sqrt{{\left(x_{i}-x_{c}\right)}^{2}+{\left(y_{i}-y_{c}\right)}^{2}+{\left(z_{i}-z_{c}\right)}^{2}}$$ (1)

where $\left(u_{i},\;v_{i}\right)$ and $d_{i}$ are the pixel coordinates and depth in the depth map D, $\left(x_{i},\;y_{i},\;z_{i}\right)$ is the coordinates of the particles within *P*, $\left(c_{x},\;c_{y}\right)$ is the center of depth map D, and $f_{u}$ and $f_{v}$ are the focal length components of the camera, defined as follows:

$$f_{u}=\frac{D_{\text{width}}}{2\text{tan}\left(\frac{\mathrm{fov}}{2}\right)},\;f_{v}=\frac{D_{\text{height}}}{2\text{tan}\left(\frac{\mathrm{fov}}{2}\right)}$$ (2)

where $D_{\text{width}}$ and $D_{\text{height}}$ are the width and height of the depth map *D* in pixels; fov is the angular camera field of view in radians.

For depth map interpolation, we utilize the 2D cubic spline interpolation method to interpolate the discrete depth map *D* to obtain a smoother and continuous depth map for optical rendering.

### Light-field-based optical rendering

The proposed optical rendering method includes optical system initialization, offline light field generation, and online image rendering using Phong’s lighting model [55], as shown in Fig. 2B. For tactile sensors with biomorphic-shaped membranes, light propagation within the sensor generates highly nonlinear light fields. The calculation of light propagation should not only consider the linear paths from the light source to the target points on the membrane but also account for the nonlinear light paths that follow the membrane’s geometry.

#### Offline light field generation

We generate 2 types of light fields: the linear light field that models light propagation along straight lines and the nonlinear light field that accounts for light propagation along the curved surface of the sensor membrane, as shown in Fig. 2C.

For the linear light field, we consider the light emitted from the source light $L_{s}$ traveling in a straight line within the sensor to the target point *T* on the membrane surface, as shown in the top of Fig. 2C. The direction of the incident light at point *T* can be expressed as $\vec{L}=L_{s}-T$. This enables us to compute the corresponding linear light field ${\hat{L}}_{\text{linear}}$, which includes the direction of the incident light rays at all target points *T* located on the membrane. The collection of all target points $T\left(x,\;y,\;z\right)$, denoted as point cloud ${\hat{P}}_{t}$, is derived from the depth map D generated by the particle-based simulation, using the following inverse camera projection model:

$$x=\left(u-c_{x}\right)\frac{z}{f_{u}},\;y=\left(v-c_{y}\right)\frac{z}{f_{v}},\;z=D\left(u,v\right)$$ (3)

For the nonlinear light field ${\hat{L}}_{\text{nonlinear}}$, light propagates through the membrane; therefore, we assume that the light path follows the membrane’s surface geometry. To compute this light path, we first define a propagation plane $P_{l}$ that contains both the light source $L_{s}$ and a target surface point T, as shown in the bottom of Fig. 2C. The vector from the light source $L_{s}$ to the target point *T* is $\vec{L}=L_{s}-T$, while the surface normal $\vec{n}$ at point *T* on the curved surface $z=f\left(x,\;y\right)$ is $\vec{n}=\left(-\frac{\partial z}{\partial x},-\frac{\partial z}{\partial y},1\right)$. The normal vector ${\vec{P}}_{l}$ of the plane $P_{l}$ can be calculated using $\vec{n}$ and $\vec{L}$: ${\vec{P}}_{l}=\vec{n}\times \vec{L}$. We determine the light path by computing the intersection curve between the plane $P_{l}$ and the membrane mesh *M*. This is achieved by checking each triangular face within the mesh *M* to determine whether it intersects with $P_{l}$ and calculating the corresponding intersection points. By connecting these points, we obtain a continuous curve that represents the intersection of the membrane mesh M with the plane $P_{l}$, which also corresponds to the path of light propagation within the membrane. The direction along the path at the target point *T* is taken as the incident light direction *L*. We can compute the nonlinear light field ${\hat{L}}_{\text{nonlinear}}$ by repeating this process for all target points T on the membrane.

#### Online image rendering

With the deformed depth map *D* obtained from particles and the offline generated light field $\hat{L}$ from all light sources, we apply Phong’s reflection model [55] for optical rendering, as shown in Fig. 2D. The overall illumination intensity *I* observed at a given point $T\left(x,y,z\right)$ of the sensor elastomer is given by 3 components: ambient, diffuse, and specular light:

$$I=k_{a}i_{a}+\sum_{m\in {\hat{L}}_{s}}\left(k_{d}\left({\hat{L}}_{m}\cdot \hat{N}\right)i_{m,d}+k_{s}{\left({\hat{R}}_{m}\cdot \hat{V}\right)}^{\alpha }i_{m,s}\right)$$ (4)

$${\hat{R}}_{m}=2\left({\hat{L}}_{m}\cdot \hat{N}\right)\hat{N}-{\hat{L}}_{m}$$ (5)

where ${\hat{L}}_{s}$ is the set of light sources; ${\hat{L}}_{m}$ is the direction of incident light emitted from the given light source *m* toward the given point *T*, which has already been computed in the generated light field ${\hat{L}}_{\text{nonlinear}}$; $i_{a}$ is the intensity of the ambient light, which refers to the background image obtained using the linear light field rendering ${\hat{L}}_{\text{linear}}$ or taken from the real sensor; $i_{m,d}$ and $i_{m,s}$ are the intensities of the diffuse and specular reflections of light source *m*, respectively; $k_{a}$, $k_{d}$, $k_{s}$, and α are all reflectance properties of the surface; ${\hat{R}}_{m}$ is the direction of the reflected light; $\hat{V}$ is the direction pointing toward the camera; and $\hat{N}$ is the normalized surface normals. We follow the inverse camera projection model [Eq. (3)] to transform the deformed depth map *D* generated from the MPM to the point cloud *P* and compute the surface normals $\hat{N}$ using the discrete partial derivatives:

$$\hat{N}=\frac{\frac{\partial p}{\partial x}\cdot \frac{\partial p}{\partial y}}{\left\|\frac{\partial p}{\partial x}\cdot \frac{\partial p}{\partial y}\right\|}$$ (6)

where the partial derivatives $\frac{\partial p}{\partial x}$ and $\frac{\partial p}{\partial y}$ are computed using the Sobel edge detector over a point *p* in *P*. The color of a light source is determined by its *R*, *G*, and *B* values, with the intensity of each channel calculated individually at the target point *T* and then combined to produce the final intensity $\left(I_{R},\;I_{G},\;I_{B}\right)$. Notably, the methods discussed above assume the light source to be a point light source. However, other types of light sources, such as line or area light sources, can be discretized into multiple point light sources through uniform sampling [56]. The final light intensity at the target point *T* is obtained by linearly summing the light intensities calculated from the light fields generated by each discrete light source.

We render the undeformed membrane using the linear light field, which serves as the background (ambient light), while the deformed area is rendered using the nonlinear light field to create the foreground (diffuse and specular light). The final tactile image is obtained by overlaying the background and foreground, as shown in Fig. 2D. Notably, for existing tactile sensors, we can use the tactile image collected from the real undeformed sensor as the background, whereas for new, nonexistent tactile sensors, the image rendered using the linear light field will be utilized as the background.

### Force prediction model

**Fig. 3.**
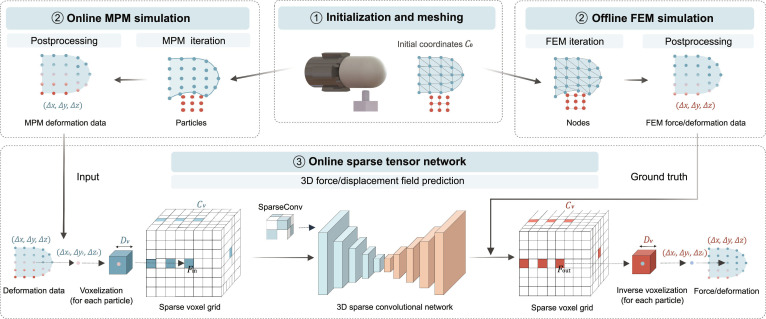
The principle of mechanical response simulation. We amplify the contact deformation and sparsify the particle density to facilitate easier understanding; an STN is used to predict force/deformation fields, where the deformation data iterated from the MPM simulation approach serve as input, while the force/deformation data computed from FEM serve as the ground truth. This framework enables rapid and accurate mechanical response simulations that approximate FEM-level precision.

Figure 3 illustrates the simulation pipeline for tactile mechanical response. After initialization and discretization, the deformation data of the sensor membrane obtained from MPM iterations are postprocessed and fed into the Sparse tensor networks (STNs) [57,58] to predict force. FEM simulation data are generated offline in advance and serve as the ground truth for training the STN. Notably, in addition to force distributions, other field data, such as deformation and stress, can also serve as network outputs for training and prediction.

STNs are advanced techniques for processing high-dimensional sparse data. Their core principle is to sparsely encode multidimensional correlations within tensors, effectively reducing storage and computational costs while preserving key features between neighboring particles, making it particularly well suited for processing particle cloud data. By leveraging STN, the sensor deformations generated by MPM can be mapped to the force/deformation distributions computed by FEM, enabling fast and accurate simulation of mechanical responses that approximate FEM-level precision.

A sparse tensor consists of a voxel coordinate matrix $C_{v}$ and an associated feature matrix ***F***. $C_{v}$ is obtained by voxelizing a particle cloud with a predefined voxel size $D_{v}$, while *F* is determined by aggregating the particle feature vectors within each voxel. The voxel size needs to be reasonably set to prevent multiple particles from falling into the same voxel, thereby preserving the particle cloud features as accurately as possible. Therefore, we first generate the initial coordinates $C_{0}\left(x_{i},\;y_{i},\;z_{i}\right)$ of the particles through meshing the sensor membrane, with the membrane in its undeformed state, and extract the field data of each particle obtained from MPM and FEM iterations. The initial particle coordinates $C_{0}$ are then voxelized with a predefined voxel size $D_{v}$ to obtain the voxel coordinates $C_{v}$. The field data–particle displacements $\left(\Delta x_{i},\;\Delta y_{i},\;\Delta z_{i}\right)$ computed by the MPM are set as input features, and the feature matrix $F_{\text{in}}$ is determined by averaging the feature values of all particles within the same voxel.

$$C_{v}=\left[\begin{array}{cccc} b^{1} & c_{x}^{1} & c_{y}^{1} & c_{z}^{1}\\ \vdots & \vdots & \vdots & \vdots \\ b^{N} & c_{x}^{N} & c_{y}^{N} & c_{z}^{N} \end{array}\right],\;F_{\text{in}}=\left[\begin{array}{ccc} f_{x}^{1} & f_{y}^{1} & f_{z}^{1}\\ \vdots & \vdots & \vdots \\ f_{x}^{N} & f_{y}^{N} & f_{z}^{N} \end{array}\right]$$ (7)

where $b^{i}$ is the batch index for particle *i*, $\left\{c_{x}^{i},\;c_{y}^{i},\;c_{z}^{i}\right\}\in {\mathbb{Z}}^{3}$ are the voxelized coordinates, and $\left\{f_{x}^{i},\;f_{y}^{i},\;f_{z}^{i}\right\}\in {\mathbb{R}}^{3}$ represent the features. The index $i\in \left[1,N\right]$, where *N* is the number of voxels after voxelization, which depends on the voxel size $D_{v}$.

The sparse convolution takes a sparse tensor as input and also produces a sparse tensor as output. In this case, the input and output share the same coordinate matrix, meaning $C_{v}^{\text{in}}=C_{v}^{\text{out}}$. The feature vector $f^{\text{out}}$ for an output coordinate *c* is then computed using the following formula:

$$f_{c}^{\text{out}}=\sum_{s\in 𝒩\left(c,K\right)}W_{s}f_{c+s}^{\text{in}},\;f_{c}^{\text{out}}\in F^{\text{out}},\;c\in C_{v}^{\text{out}}$$ (8)

where *s* represents the offset used to locate the corresponding input coordinates within the *c*-centered neighborhood covered by the kernel size *K*, denoted as N(c,K). $f_{c+s}^{\text{in}}$ denotes the input feature vector at the input coordinate c+s, while $W_{s}$ represents the coefficient learned during the training process. Using the coordinate matrix $C_{v}^{\text{out}}$ and the feature matrix $F^{\text{out}}$, the output sparse tensor can be generated.

The bottom of Fig. 3 illustrates the overall structure of the proposed network. We first voxelize the particle cloud and convert the particle displacements into a sparse tensor. The network is based on the Minkowski UNet14 architecture [58], which follows a symmetric encoder–decoder structure. Both the input and output consist of 3-channel field data corresponding to the *X*, *Y*, and *Z* directions. The encoder extracts features from the sparse tensor, progressively reducing the number of feature vectors while increasing the channel dimensions. Conversely, the decoder decreases the channel dimensions while restoring the number of feature vectors. After passing through the output layer, the feature vectors have a shape of *N* × 3, where *N* represents the number of voxels and 3 denotes the feature dimensionality. Finally, we perform inverse voxelization on the output sparse tensor to obtain the particle force/deformation.

The network is trained using the L1 loss (mean absolute error [MAE]) function and optimized with the Adam optimizer. The dataset comprises 10,780 samples, with 8,624 samples from 10 seen indenters used for training (25% reserved for validation) and 2,156 samples from 4 unseen indenters used exclusively to test the model’s generalization performance. Training is performed with a batch size of 32, an initial learning rate of 1 × 10^−3^, and a learning rate decay factor of 10 every 10 epochs. The final model is saved after 100 epochs of training.

### Experimental setup

We evaluate SimTac across 4 key aspects:

• Accuracy: We evaluate the performance of SimTac by comparing the simulation results with ground truth data. Quantitative accuracy is assessed under different conditions, including contact with objects of various sizes, shapes, and textures, as well as different contact positions, postures, and motions.
• Efficiency: We evaluate the runtime performance of each module within the simulator to evaluate its computational efficiency, which is vital for tactile-based real-time simulations that require extensive iterations.
• Flexibility: We evaluate the flexibility of SimTac by simulating tactile sensors with different shapes and material parameters, which is crucial for simulating biomorphic-shaped tactile sensors, rather than being limited to sensors with fixed shapes and materials.
• Applicability: We evaluate the applicability of SimTac from 2 main perspectives: sensor prototyping and tactile-based Sim2Real tasks. For sensor prototyping, we first design sensor prototypes within SimTac and then fabricate the corresponding real-world sensors, using an elephant-trunk-shaped sensor as a representative example. For perception tasks, we assess SimTac’s performance across several tactile-based Sim2Real tasks, including object classification, slip detection, and contact safety assessment, using a finger-shaped sensor as the test case.

**Fig. 4.**
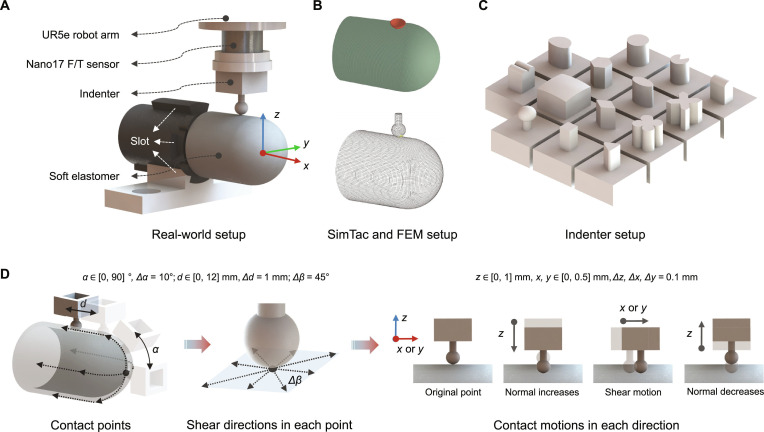
Data collection setup on the finger-shaped GelTip sensor. (A) Real-world data collection platform, capable of synchronously capturing tactile images and contact forces, with the indenter’s contact posture controlled by the robotic arm. (B) Configuration in SimTac and FEM replicated from the real-world scenario. (C) Indenters with different shapes used in the experiment. (D) The movement trajectory of the indenter during the data collection process. The contact points cover most of the sensor’s surface, including a combination of vertical and tangential motion.

We used the finger-like tactile sensor GelTip [10] for the evaluation of accuracy and efficiency. GelTip closely resembles a human finger in size and shape, featuring high curvature and an omnidirectional sensing surface. Compared to flat sensors, it exhibits more complex deformations and internal light fields, making the simulation more challenging. Meanwhile, compared to other biomorphic shapes, its more uniform and regular geometry simplifies the design of data collection trajectories for the corresponding real sensor. Two types of GelTip sensors were configured: one without markers, used to collect tactile images for optical response comparison, and the other with markers on the skin (where the motion of the markers indicates the deformation of the sensor skin), used to collect images with marker motions and total contact force for mechanical response comparison (refer to Fig. 1B, shape and pattern input; markers have been added to the sensor membrane). To collect tactile data with the GelTip sensor, we secured the GelTip in place and mounted the indenter along with the ATI Nano17 force/torque sensor onto a UR5e robotic arm. We controlled the robotic arm to facilitate contacts between the indenter and the GelTip at various positions and orientations. Tactile data were collected using 14 different indenter shapes, covering different contact positions, depths, and shear motions. Further details can be found in Fig. 4 and Note S4.2.

## Results

### Accuracy evaluation

This evaluation focuses on 2 key aspects: optical response simulation performance and mechanical response simulation performance. For the evaluation of optical response, we use tactile images collected from real-world sensors as references. For the evaluation of mechanical response, we adopt 2 reference sources: dense deformation and force fields generated by FEM, as well as tactile images with sparse marker motion and total force measurements collected from real sensors.

#### Optical response simulation performance

**Fig. 5.**
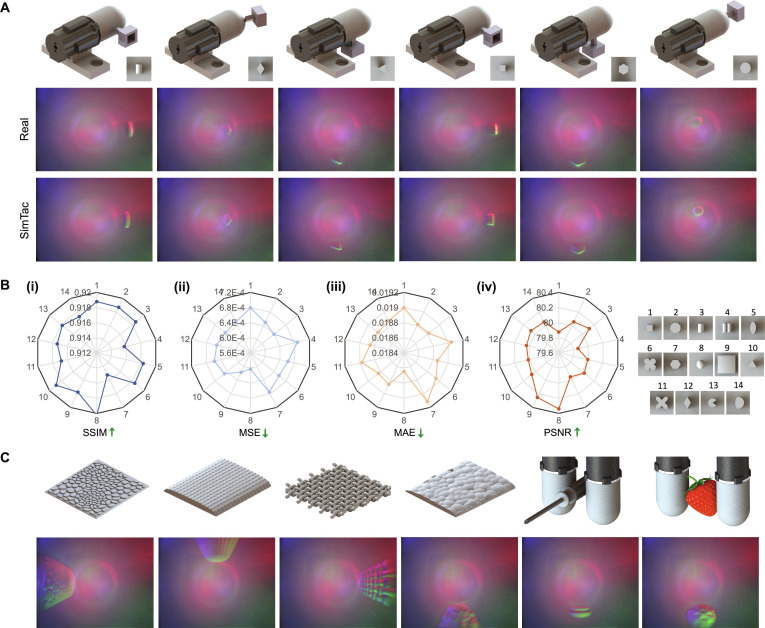
The performance evaluation of optical response simulation. (A) The comparison of tactile images collected from the real world and those generated from the proposed simulator under varying contact positions and indenter shapes. (B) The quantitative analysis results of the optical simulation under contact with different indenters, evaluated using (i) SSIM, (ii) MSE, (iii) MAE, and (iv) PSNR. (C) The optical simulation results when the sensor interacts with different textures.

We followed the experimental setup and data collection methods outlined in Fig. 4 and Note S4.2, collecting 6,538 data pairs in both the simulation and real-world environments to evaluate the accuracy of the simulator. Figure 5A compares the real tactile images with their corresponding simulated images for various indenter shapes, contact positions, and orientations. The results demonstrate that the simulated images accurately reproduce the shape distortion caused by the deformation area being close to the wide-angle lens and successfully simulate the deformation of the contact area, the color distribution of reflected light, and the formation of specular highlights. To quantitatively assess the similarity between simulated and real tactile images, we use 4 widely used image similarity metrics: structural similarity index (SSIM) [59], mean squared error (MSE), MAE, and peak signal-to-noise ratio (PSNR) [59]. Figure 5B presents the distribution of these metrics, where the simulated tactile images show high similarity and low pixel error compared to the corresponding real tactile images across all indenter shapes and contact poses (more details can be seen in Fig. S13). Moreover, our simulator is capable of optically simulating fine surface textures and real object contacts. Figure 5C showcases textured surfaces of various types, along with objects from the YCB dataset [60], which feature more intricate contact details and larger object sizes compared to the indenters (more detailed simulations of fine texture are provided in Note S4.8). Further comparisons of optical response between SimTac and other simulators can be found in Note S4.6.2.

#### Mechanical response simulation performance

**Fig. 6.**
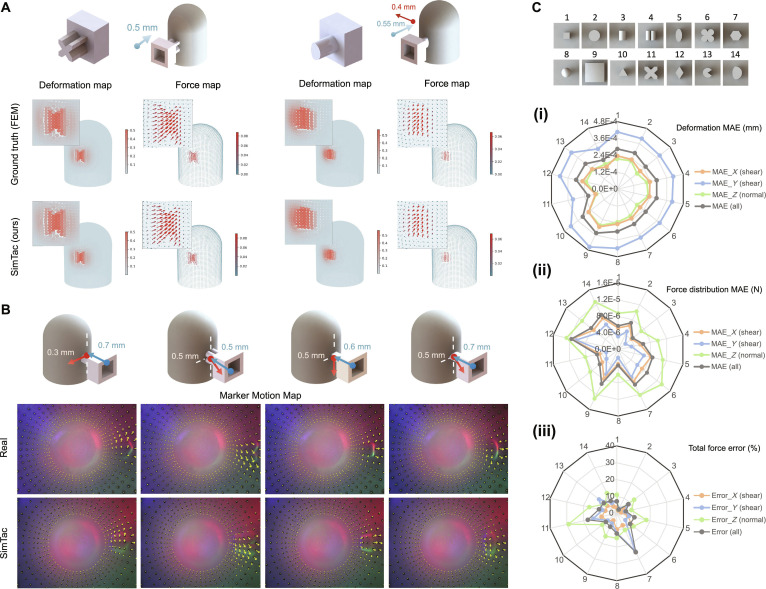
The performance evaluation of mechanical response simulation. (A) Comparison of 3D deformation and force fields between SimTac simulation and ground truth from FEM on the test set across different contact scenarios. (B) Comparison of marker motion between the SimTac simulation and ground truth from the real world across different contact scenarios. (C) The quantitative analysis results of the mechanical response simulation when in contact with different indenters. (i) MAE of deformation field; (ii) MAE of force field; (iii) percentage error of the total force.

Following the experimental setup and data collection methods outlined in Fig. 4 and Note S4.2, we collected 10,780 pairs of tactile data using both the particle-based simulation approach and FEM, with data from 10 seen objects used for model training (Fig. 6C, indenters 1 to 10) and data from 4 unseen objects used for testing (Fig. 6C, indenters 11 to 14). Accuracy is evaluated on the dataset collected from all indenters. For the dense reference data from FEM, we performed direct comparison, while for the sparse reference data from reality, we converted the dense fields into sparse fields through downsampling deformation field data or summing force field data to calculate the total force before comparison.

We first evaluate the dense field results, as shown in Fig. 6A, heatmaps are utilized to visualize the comparison between the deformation/force fields generated from SimTac and the ground truth computed via FEM on unseen indenters (see more results in Fig. S14). The results demonstrate that the neural network achieves highly accurate particle-level predictions of dense deformation/force fields in both normal and tangential directions. The simulated field data exhibit excellent smoothness and continuity, driven by the neural network’s ability to capture nonlinear relationships between adjacent particle features and effectively extract key local characteristics. To further quantify the accuracy, we compute the MAE between the SimTac-generated fields and the FEM-computed fields across the entire dataset, as shown in Fig. 6C(i) and (ii). SimTac achieves high accuracy in simulating dense deformation and force field, with test set MAEs of 2.77 × 10^−4^ mm (deformation) and 8.6 × 10^−6^ N (force), averaged over the *X*, *Y*, and *Z* directions. Across the entire dataset, the MAEs are 2.84 × 10^−4^ mm and 7.4 × 10^−6^ N, demonstrating strong predictive performance and generalization to diverse object shapes and poses in sensor contact scenarios (a detailed quantitative analysis can be found in Fig. S15).

To evaluate the sparse displacement field, we downsample the simulated dense deformation field to match the sparsity of the markers from the real sensor. The downsampled field is then projected into the camera coordinate system to obtain the corresponding marker positions. These markers are overlaid onto the rendered tactile images, as shown in Fig. 6B. This figure illustrates the comparison between simulated and real-world results for various indenters under different contact conditions, where the yellow arrows indicate the direction and magnitude of the marker motions. To evaluate the sparse force data, we aggregate the simulated force field along the *X*, *Y*, and *Z* axes to compute the total force. The accuracy is assessed by calculating the MAE between the simulated and real-world force measurements, shown in Fig. 6C(iii). The MAE between the predicted total force and the real one across the entire dataset is 0.021 N (13.18% of the actual force) in the *X* direction (shear direction), 0.013 N (9.24%) in the *Y* direction (shear direction), and 0.134 N (6.27%) in the *Z* direction (normal direction). Notably, the normal force exhibits a much larger magnitude compared to the shear force, resulting in correspondingly larger prediction errors. Further comparisons of mechanical response between SimTac and other methods can be found in Note S4.6.1.

### Efficiency of SimTac

We evaluated the efficiency of SimTac by measuring the online time consumption of each module, including sensor deformation iteration, optical response simulation, and mechanical response simulation, all deployed to simulate the finger-like tactile sensor GelTip and run on the graphics processing unit (GPU). All simulations were conducted on an Ubuntu 20.04 system with an i7-13700HX 16-core processor and an NVIDIA GeForce RTX 4060 GPU. The performance of the particle-based deformation simulation was evaluated under 3 different input particle quantities (40,000, 300,000, and 1.3 million), resulting in frame rates of 250, 33, and 10 frames/s (FPS), respectively. The optical rendering performance was assessed at 3 output image resolutions (320 × 240, 640 × 480, and 1,280 × 960), yielding frame rates of 100, 25, and 10 FPS, respectively. The neural-network-based force/deformation prediction efficiency was tested with 3 point cloud sizes (1,000, 5,000, and 25,000), achieving frame rates of 100, 76, and 62 FPS, respectively.

### Flexibility of SimTac simulator

For vision-based tactile sensors, the optical response is primarily influenced by the sensor shape, camera parameters, and internal light sources, while the mechanical response is mainly affected by the material properties of the sensor membrane. To evaluate the flexibility of the proposed simulator, we first validate its optical response on tactile sensors with different optical setups, especially for those tactile sensors with biomorphic shapes, and then verify its mechanical response with varying material parameters.

**Fig. 7.**
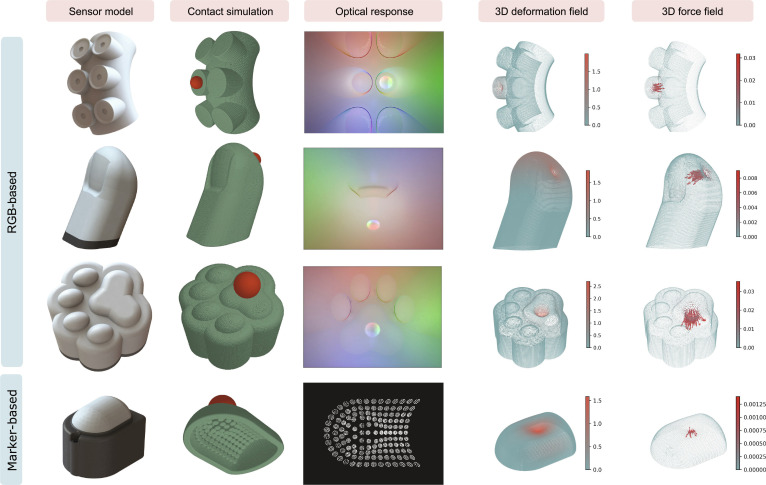
The flexibility of the SimTac simulator. The proposed simulator can be applied to tactile sensors with diverse biomorphic shapes and is also capable of simulating both RGB-based and marker-based tactile sensors.

To evaluate the flexibility of SimTac across different sensor configurations, we designed tactile sensors with diverse shapes, sizes, and optical setups, including variations in LED placements and quantities, as well as camera positions and lens angles. Specifically, our study features 4 types of tactile sensors: 3 novel biomorphic tactile sensors modeled after the shape of an octopus tentacle, a human thumb and a cat’s paw, and a curved, marker-based DigiTac sensor [61]. Figure 7 shows the simulation results of tactile feedback during contact with a spherical object, including contact deformation, optical response, and mechanical response (with further details in Fig. S16). Our approach can be applied to the simulation of biomorphic-shaped tactile sensors, offering potential applications in the development of biomorphic soft robots. Moreover, for the marker-based DigiTac sensor, our method can simulate the motion of physical pins [61] during contact. In Note S4.7, we provide an example of how the mechanical response prediction model is adapted to sensors with new geometries using a transfer learning approach.

**Fig. 8.**
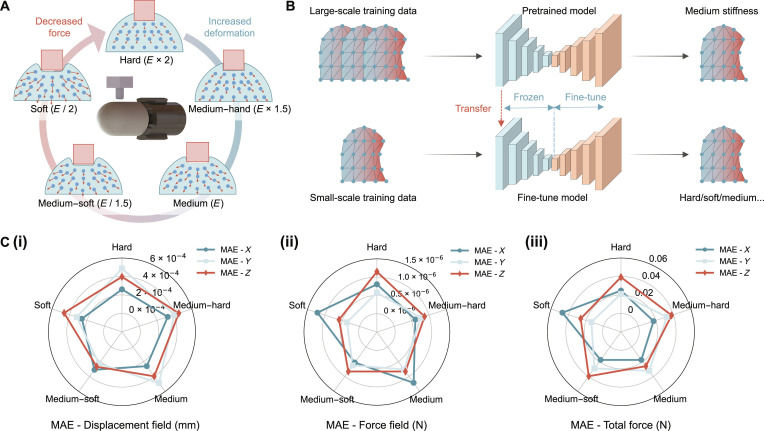
The flexibility of the SimTac simulator. (A) Deformation of membranes with varying hardness under identical contact conditions. Softer membranes exhibit larger deformation and lower force. (B) The trained network can be applied to sensor membranes of different materials through a fine-tuning process, requiring only a small additional FEM ground-truth dataset. (C) The MAE of (i) deformation field, (ii) force field, and (iii) total force in the *X*, *Y*, and *Z* directions between the simulated and ground-truth values, evaluated across GelTip sensor membranes of different materials.

To evaluate the flexibility of SimTac on sensor skins with different material properties, we modified the original Young’s modulus of the sensor membrane (0.145 MPa, medium), doubling it (0.29 MPa, hard) to create a stiffer contact surface and halving it (0.0725 MPa, soft) to create a softer one (Fig. 8A). For each stiffness variation, a limited set of ground truth data was collected from FEM simulations to fine-tune the pretrained neural network (trained using data with medium stiffness), where only the decoder is updated while the encoder remains fixed (Fig. 8B). We then evaluate the fine-tuned neural network on membranes of corresponding stiffness, using MAE to measure the deviation between simulated results and ground truth. As visualized in the radar chart in Fig. 8C, the MAE for the deformation field remains below 4.37 × 10^−4^ mm, the MAE for the force field stays under 1.16 × 10^−6^ N, and the MAE for total force is also within 0.042 N across membranes ranging from soft to stiff. These results demonstrate that the mechanical response simulation method can be applied effectively to sensors with different material parameters through the fine-tuning strategy.

### Application of SimTac

We first demonstrate SimTac’s sensor prototyping capability by designing a biomorphic-shaped tactile sensor in simulation, with an elephant trunk shape as an example, and then transferring the design to the real world to fabricate the corresponding physical sensors. We further evaluated SimTac’s applicability in 3 tactile-guided Sim2Real tasks using a human finger-shaped tactile sensor: object classification, slip detection, and contact safety assessment. These tasks reflect key aspects of biological tactile perception, enabling the interpretation of object properties, monitoring of dynamic interactions, and avoidance of harmful contact. For the object classification task, the simulated optical response was utilized to infer the shapes of contacted objects. In the slip detection task, both the simulated optical responses and mechanical responses were used to determine whether an object was slipping relative to the sensor surface. For contact safety assessment, the simulated force fields were used to evaluate the risk of damaging the sensor skin.

#### Simulation and fabrication of an elephant-trunk-shaped tactile sensor

**Fig. 9.**
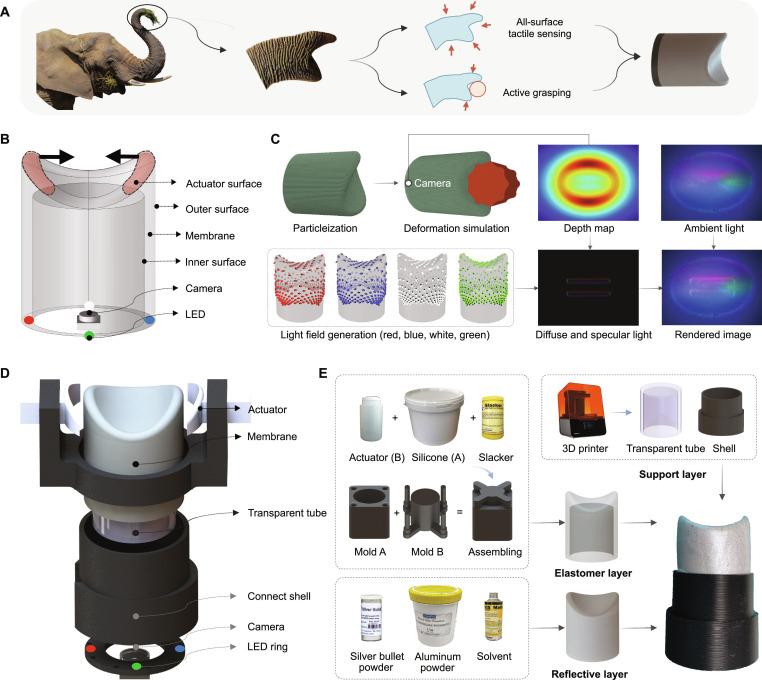
Simulation and fabrication of a biomorphic-elephant-trunk-shaped tactile sensor. (A) The inspiration from the elephant trunk, which enables all-surface tactile sensing and active grasping capability. (B) Overview of the sensor prototype in simulation. (C) Simulation pipeline of the sensor, involving mesh particleization, deformation modeling, and synthetic tactile image generation through light field rendering. (D) Exploded view of the real sensor design, showing its modular components, including an actuator, sensor membrane, transparent tube, and integrated optical system. (E) Fabrication workflow for the sensor, including mold-based elastomer casting, structural 3D printing, and reflective layer coating.

As shown in Fig. 9A, the tip of an elephant’s trunk features 2 finger-like protrusions that can grasp and manipulate objects such as pincers. Its entire surface is covered with skin, providing rich tactile feedback. In contrast, human fingers cannot naturally form a pincer-like structure with a large tactile surface area; instead, they must work together with the palm to achieve similar grasping capabilities. Inspired by the grasping mechanism and advanced tactile sensing of an elephant’s trunk, we designed a biomorphic sensor based on its morphology, where flexible protrusions close inward to execute grasping actions. As illustrated in Fig. 9B, we first designed a silicone membrane in the shape of an elephant trunk, specified the parameters of the LEDs and cameras, and defined the actuation surface along with the contact conditions between the sensor and the object. We then used SimTac to generate tactile feedback when the sensor interacted with objects, allowing us to evaluate whether the tactile images could accurately and clearly reflect the contact state. On the basis of the parameter settings in the simulation, we subsequently fabricated a physical prototype of the sensor, as shown in Fig. 9D, following the manufacturing process illustrated in Fig. 9E (the detailed parameters of the sensor are provided in Note S4.3).

**Fig. 10.**
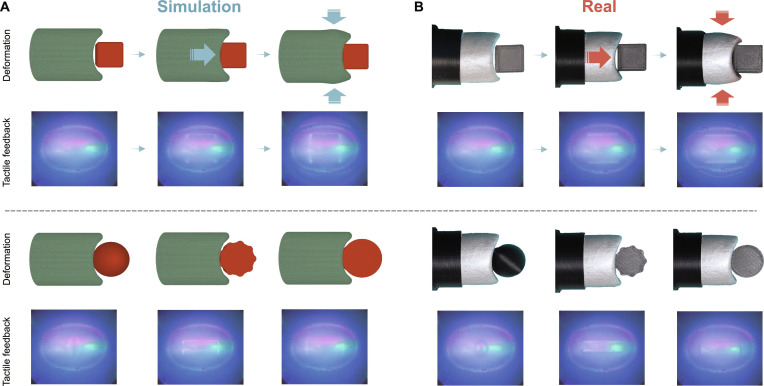
Comparison between (A) the simulated and (B) the real sensor deformation and tactile perception of a biomorphic-elephant-trunk-shaped tactile sensor.

Figure 10 presents the deformation of both the simulated and real elephant trunk when interacting with objects. The trunk first senses the object’s shape through vertical contact and then performs a grasping action by closing its protrusions. The tactile feedback throughout this process is also shown, where the optical response reveals crucial information such as the object’s position, shape, and the sensor’s active deformation during grasping. The experimental results demonstrate that SimTac enables the design of innovative biomorphic mechanisms with tactile sensing capabilities by leveraging the interaction mechanisms between biological organisms and their environments. With its capability to simulate tactile responses for diverse biomorphic structures, SimTac holds substantial potential for simulating and designing advanced biomorphic sensors and even grippers that integrate both active deformation and high-resolution tactile sensing.

#### Sim2Real transfer of contact perception

**Fig. 11.**
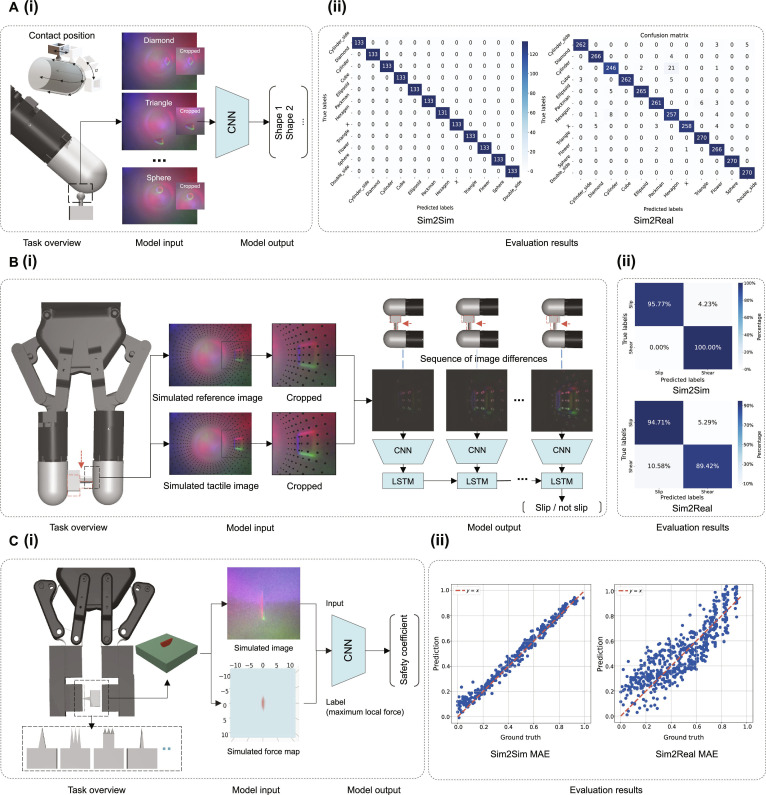
Performance evaluation of the tactile-based Sim2Real tasks. (A) (i) Pipeline and (ii) results of the object classification Sim2Real task. (B) (i) Pipeline and (ii) results of the slip detection Sim2Real task. (C) (i) Pipeline and (ii) results of the contact safety assessment Sim2Real task. Convolutional neural network (CNN) is used as the encoder in the models.

The overview of this task is shown in Fig. 11A(i). We used a classification model using the ResNet50 architecture [62], which took tactile images as input and output the probabilities for each shape class (the detailed model structure is provided in Fig. S18A). We established 2 experimental groups, i.e., Sim2Sim and Sim2Real, to evaluate the model’s performance, with the model trained on synthetic data, tested on a synthetic test set in the Sim2Sim group, and assessed on real-world data with zero-shot transfer in the Sim2Real group (detailed in Note S4.4).

As shown in Fig. 11A(ii), the Sim2Sim scenario achieved the highest classification accuracy of 100%, while the Sim2Real scenario reached an accuracy of 97.0%. In the Sim2Real scenario, classification failures primarily occurred with indenters of similar shapes. Insufficient contact between the flat indenter and the curved membrane, combined with shape distortion caused by the wide-angle lens, led to the emergence of similar features in the contact area, which prevented the full representation of the indenters’ geometric characteristics and resulted in misclassification. Failure cases can be found in Fig. S17B.

#### Sim2Real transfer of slip detection

For slip detection tasks, collecting tactile data from real-world scenarios poses substantial risks, especially when sensors are exposed to sharp objects or experience prolonged wear. This can damage or even scratch the sensor membrane due to its thin and fragile reflective layer. Therefore, simulating object slip on the sensor surface and training slip detection models with simulated data, while ensuring effective Sim2Real transfer, is essential. The overview of this task is shown in Fig. 11B(i). We proposed a deep-neural-network-based approach to detecting slip, which takes a sequence of 8 tactile images as input and outputs a binary classification indicating whether slip has occurred or not (the detailed model structure is provided in Fig. S17B). Two experimental groups were established, i.e., Sim2Sim and Sim2Real, to evaluate the model’s performance using synthetic and real data, respectively (detailed in Note S4.4).

Figure S18A presents an example of input image sequences showing both slip and nonslip events in simulated and real-world data. The image sequence demonstrates that the key difference between slip and nonslip lies in the relative motion between the object and the sensor surface. In nonslip cases, the object’s contour and surface markers move synchronously, while in slip cases, the object continues to move as the markers remain nearly stationary, indicating relative motion. The model performance is shown in Fig. 11B(ii), the prediction accuracy of the Sim2Sim group was 97.89%, while the Sim2Real group achieved an accuracy of 92.06%. Additional results on Sim2Real slip detection under dynamic interactions can be found in Note S4.9.

#### Sim2Real transfer of contact safety assessment

Biological organisms rely on tactile perception of local force distribution to assess the safety of physical contact with their environment. For instance, human hands, cat paw pads, and octopus tentacles can detect concentrated pressure when touching sharp objects, triggering rapid withdrawal or other protective responses. This ability is crucial for ensuring safe interactions in robotics as well. However, collecting such tactile data in real-world scenarios carries substantial risks. To mitigate these challenges, achieving robust Sim2Real transfer is essential for developing safer and more reliable robotic tactile sensing systems. The overview of this task is shown in Fig. 11C(i). We constructed a regression model using the ResNet50 architecture [62], which takes tactile images as input and predicts the safety coefficient, a metric used to quantify the magnitude of local contact pressure, ranging from 1 (low local pressure, safe contact) to 0 (high local pressure, high-risk contact). We established 2 experimental groups to evaluate the performance of the model trained on synthetic data (Sim2Sim) and transferred with zero-shot to real-world scenarios (Sim2Real), detailed in Note S4.4.

Figure 11C(ii) shows the performance of the trained model in assessing contact safety, achieving an MAE of 0.028 between predicted and ground-truth safety coefficients in Sim2Sim evaluations. In the zero-shot Sim2Real evaluation, the model’s MAE was 0.105. When deployed on a real sensor, the simulation-trained model effectively captures the increasing risk of damage as the indentation depth increases, demonstrating its high value for robotic self-protection. The higher Sim2Real error compared to Sim2Sim can be attributed to several factors, as shown in Fig. S19B: First, 3D-printed objects exhibit surface textures absent in simulations, introducing artifacts that affect prediction error. Second, as the indentation depth increases, the deformation of the silicone surface alters light distribution, causing the overall image to darken. Third, some real-world data suffer from insufficient indentation, resulting in tactile images that only capture partial contact contours rather than the full contact area. Finally, certain contact poses may cause objects to extend beyond the camera’s field of view, limiting the completeness of tactile information.

## Discussion

We introduce SimTac, a physics-based simulator for biomorphic-vision-based tactile sensors that can generate synthetic tactile images along with corresponding dense deformation and force fields. The accuracy of SimTac is validated by comparing its outputs with real-world optical and mechanical responses. SimTac demonstrates remarkable flexibility, supporting a wide range of biomorphic geometries, materials, and optical configurations while maintaining high simulation efficiency across varying particle densities and image resolutions. To further demonstrate its capabilities, we design a prototype of a biomorphic elephant trunk sensor through simulation and then fabricate a real sensor based on this design. By comparing the tactile responses, we validate SimTac’s powerful ability to simulate new biomimetic-shaped sensors. This field remains largely unexplored, yet SimTac offers valuable new insights and support for the design and simulation of biomimetic soft robotic tactile systems. Moreover, SimTac shows promising zero-shot sim-to-real transfer performance in representative tactile tasks, including object shape classification, slip detection, and contact safety assessment. These tasks are crucial for enhancing a robot’s ability to perceive object properties, strengthen its interaction with the environment, and protect it from potential harm.

In general, tactile images can provide rich information about the texture, shape, and pose of the object in contact, which are crucial for identifying its properties. Further deformation and force fields can offer detailed contact information, which is vital for better object interaction. With increasing task complexity, tactile sensors have progressed from simple cubic and hemispherical geometries to finger-like and biomorphic designs. The emergence of such complex morphologies introduces greater challenges for modeling and data generation. This development highlights the need for efficient and accurate simulators capable of producing tactile data across a diverse range of sensor architectures.

For deformation simulation, traditional depth map smoothing methods overlook the physical properties of the membrane, while FEM struggles to generate high-density deformation data in real time. In contrast, combining a particle-based iteration approach with neural networks provides a more efficient approach: The particle-based approach simulates membrane deformation, while neural networks can quickly map this deformation to force fields computed offline by FEM, enabling efficient and accurate generation of dense field data. For optical rendering, data-driven and generative methods typically rely on real sensor data and have yet to be applied to the simulation of complex-shaped sensors. In comparison, physics-based rendering methods, such as path tracing, require more rendering time and are less efficient. The light-field-based lighting model rendering method, however, simulates the propagation of light within the sensor membrane of a specific shape, substantially accelerating rendering computations when deployed on a GPU.

The proposed simulator also has some limitations. The training of the neural network relies on the collection of FEM ground-truth data. Although we can fine-tune the predictive model by collecting small batches of FEM data for sensors with the same shape but different materials, data collection for entirely new shapes of sensors still takes a few days at high mesh densities, although this process is conducted offline and can be accelerated using a GPU. (We also present results demonstrating the transfer of the pretrained model to sensors with new geometries via fine-tuning; please refer to Note S4.7 for more details.)

Overall, our proposed simulator can be applied to tactile sensors with various biomorphic shapes and material parameters. By recording the indices of all particles during particleization, we can control any particle’s motion and achieve active deformation in arbitrary regions, offering potential for simulating actuators. This flexibility eliminates the constraints of fixed sensor geometries, allowing for the integration of biomorphic designs inspired by nature. Therefore, it expands the functionality and application range of tactile sensors, with the potential to drive the development of new robots equipped with tactile sensing.

## Data Availability

Please contact the authors to obtain the data upon reasonable request.

## Supplementary Materials

Supplementary 1 Supplementary Notes Tables S1 to S6 Figs. S12 to S25 Movies S1 to S6
