# Supplementary material for: SimTac: A Physics-Based Simulator for Vision-Based Tactile Sensing with Biomorphic Structures
Source: Cyborg Bionic Syst. 2026 Feb 24;7:0510. doi: 10.34133/cbsystems.0510 (PMC12929814; doi:10.34133/cbsystems.0510)
Supplement: Supplementary 1 — Supplementary Notes Tables S1 to S6 Figs. S12 to S25 Movies S1 to S6 [file cbsystems.0510.f1.zip › Figure 14.pdf]

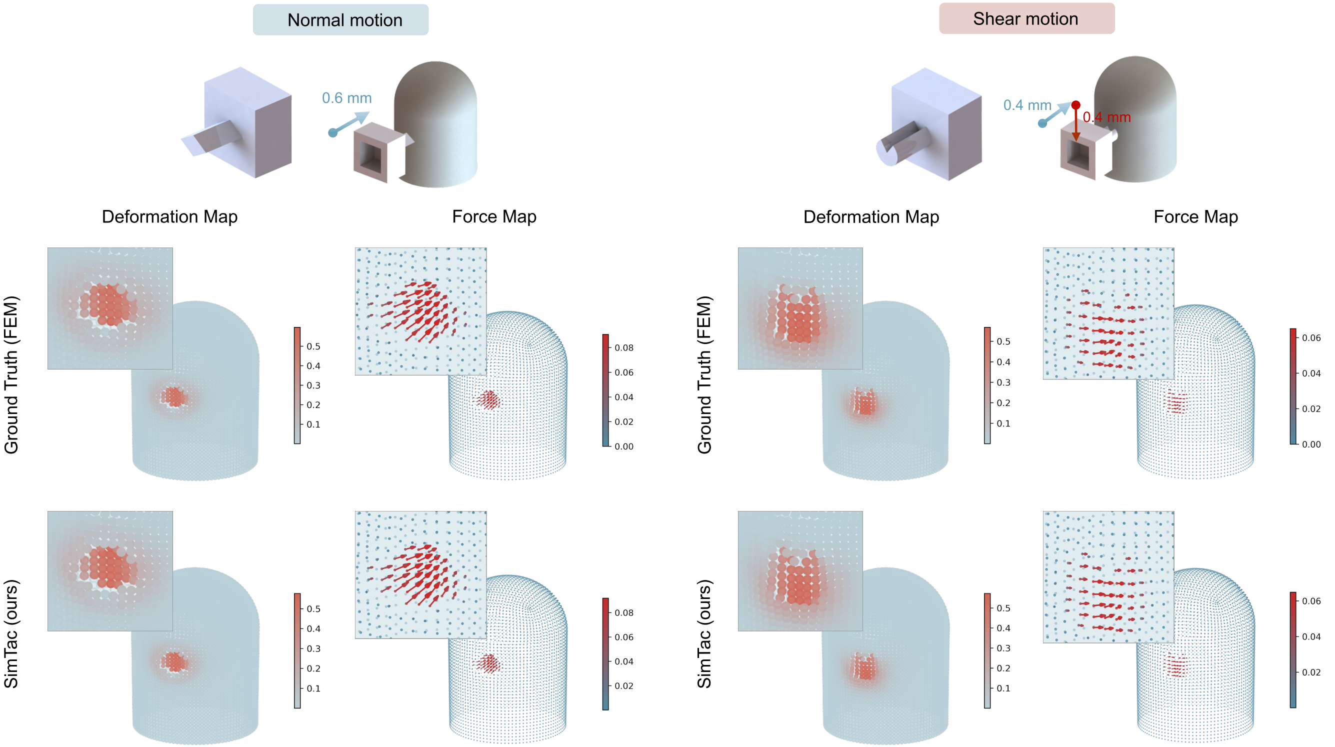
Normal motion
Shear motion
0.6 mm
0.4 mm
0.4 mm
Deformation Map
Force Map
Deformation Map
Force Map
Ground Truth (FEM)
SimTac (ours)
Ground Truth (FEM)
SimTac (ours)
0.5
0.4
0.3
0.2
0.1
0.08
0.06
0.04
0.02
0.00
0.06
0.04
0.02
0.00
