# Supplementary material for: SimTac: A Physics-Based Simulator for Vision-Based Tactile Sensing with Biomorphic Structures
Source: Cyborg Bionic Syst. 2026 Feb 24;7:0510. doi: 10.34133/cbsystems.0510 (PMC12929814; doi:10.34133/cbsystems.0510)
Supplement: Supplementary 1 — Supplementary Notes Tables S1 to S6 Figs. S12 to S25 Movies S1 to S6 [file cbsystems.0510.f1.zip › Figure 20.pdf]

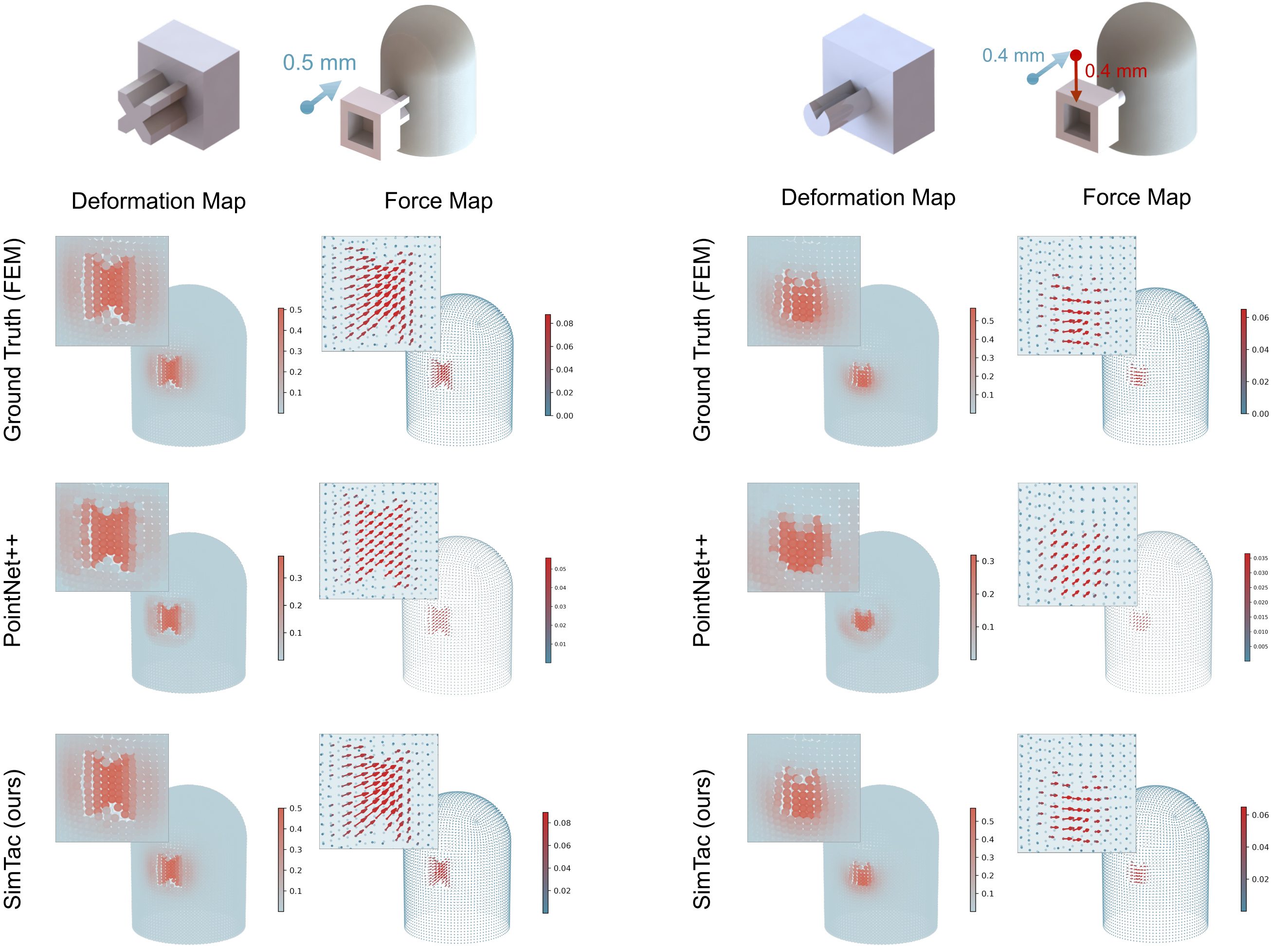
0.5 mm
0.4 mm
0.4 mm
Deformation Map
Force Map
Deformation Map
Force Map
Ground Truth (FEM)
PointNet++
SimTac (ours)
Ground Truth (FEM)
PointNet++
SimTac (ours)
