# Supplementary material for: SimTac: A Physics-Based Simulator for Vision-Based Tactile Sensing with Biomorphic Structures
Source: Cyborg Bionic Syst. 2026 Feb 24;7:0510. doi: 10.34133/cbsystems.0510 (PMC12929814; doi:10.34133/cbsystems.0510)
Supplement: Supplementary 1 — Supplementary Notes Tables S1 to S6 Figs. S12 to S25 Movies S1 to S6 [file cbsystems.0510.f1.zip › Figure 22.pdf]

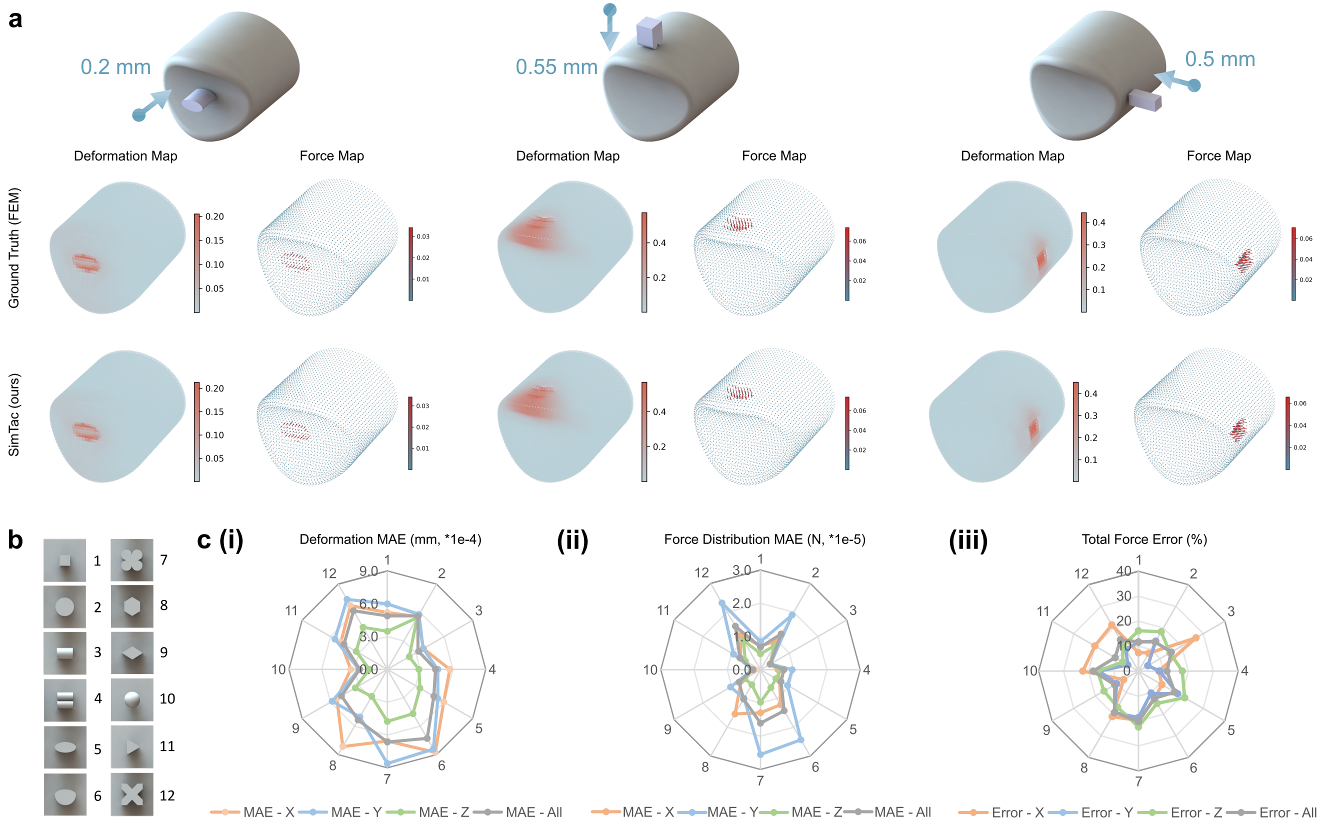

a
0.2 mm
0.55 mm
0.5 mm
Deformation Map
Force Map
Ground Truth (FEM)
SimTac (ours)
b
c (i)
Deformation MAE (mm, *1e-4)
(ii)
Force Distribution MAE (N, *1e-5)
(iii)
Total Force Error (%)
MAE - X
MAE - Y
MAE - Z
MAE - All
Error - X
Error - Y
Error - Z
Error - All
