# Supplementary material for: SimTac: A Physics-Based Simulator for Vision-Based Tactile Sensing with Biomorphic Structures
Source: Cyborg Bionic Syst. 2026 Feb 24;7:0510. doi: 10.34133/cbsystems.0510 (PMC12929814; doi:10.34133/cbsystems.0510)
Supplement: Supplementary 1 — Supplementary Notes Tables S1 to S6 Figs. S12 to S25 Movies S1 to S6 [file cbsystems.0510.f1.zip › Figure 24.pdf]

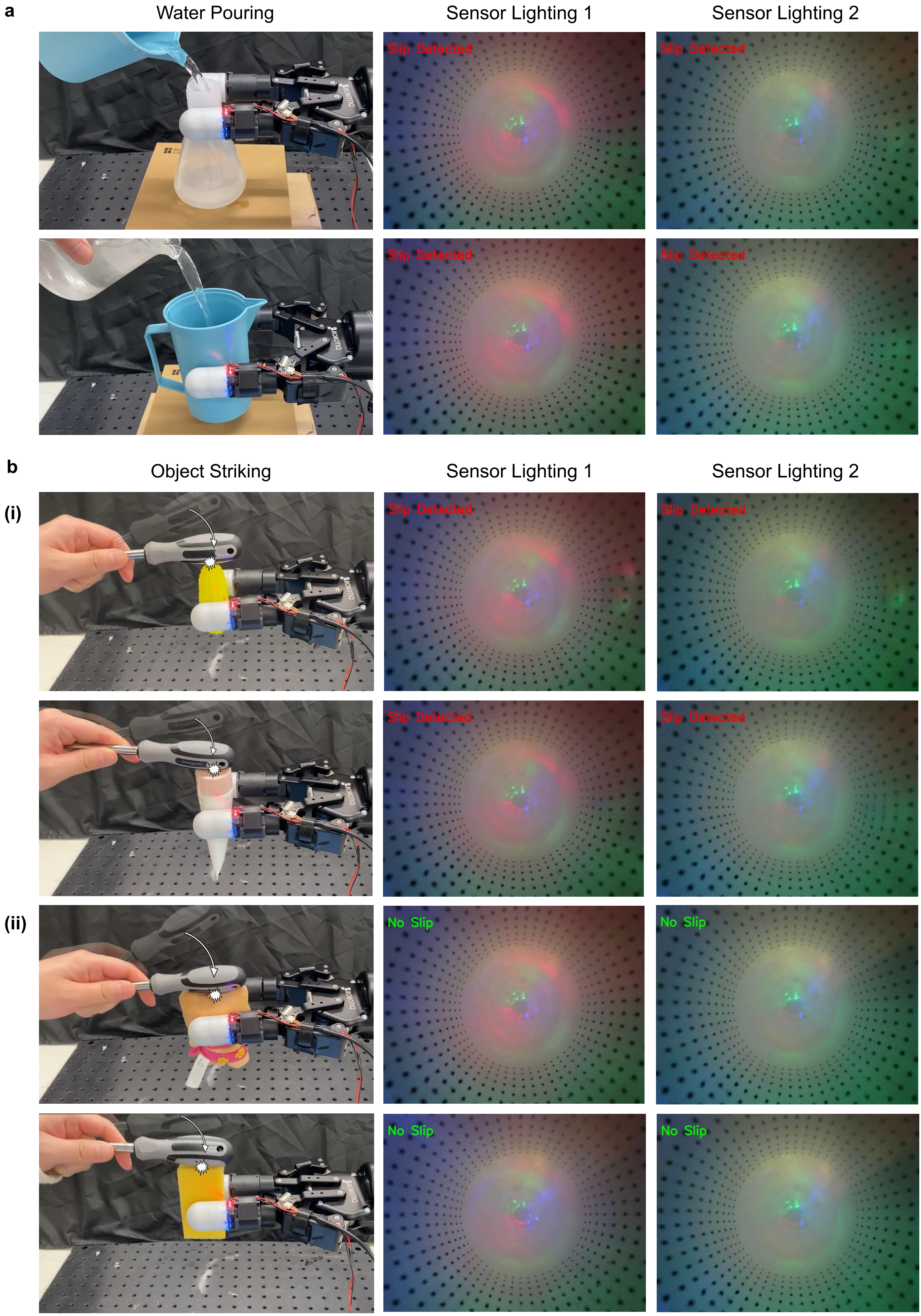

a
Water Pouring
Sensor Lighting 1
Sensor Lighting 2
Slip Detected
Slip Detected
Slip Detected
Slip Detected
b
Object Striking
Sensor Lighting 1
Sensor Lighting 2
(i)
Slip Detected
Slip Detected
Slip Detected
Slip Detected
(ii)
No Slip
No Slip
No Slip
No Slip
ROBOTIQ
