# Supplementary material for: SimTac: A Physics-Based Simulator for Vision-Based Tactile Sensing with Biomorphic Structures
Source: Cyborg Bionic Syst. 2026 Feb 24;7:0510. doi: 10.34133/cbsystems.0510 (PMC12929814; doi:10.34133/cbsystems.0510)
Supplement: Supplementary 1 — Supplementary Notes Tables S1 to S6 Figs. S12 to S25 Movies S1 to S6 [file cbsystems.0510.f1.zip › SimTac_Supplementary_Material.pdf]

Supplementary Materials

4.1 MPM iteration method

We employ the Material Point Method (MPM) [1], a particle-based simulation approach, to model the deformation of sensor skin upon contact with an object. MPM discretises objects into particles that carry properties, such as mass and velocity, and updates their state through an iterative process of exchanging properties between particles and virtual grids. This process enables a realistic simulation of deformation over time. The grids remain fixed, while properties are transferred between particles and grid nodes to facilitate the simulation. Each time step consists of four stages: (1) Particle-to-Grid (P2G): The particle properties are interpolated onto neighbouring grid nodes; (2) Grid Operations: momentum transferred from nearby particles are computed, and the velocities of the grid nodes are updated accordingly; (3) Grid-to-Particle (G2P): the updated velocities are interpolated back to the particles; and (4) Particle Operations: particle positions are updated, with boundary conditions applied.

4.1.1 Initialisation.

The sensor skin and object are discretised into m particles, with a virtual grid consisting of n fixed nodes to transfer the particle properties. For the p-th particle, its position is indicated as $x_p \in \mathbb{R}^3$, and its velocity is given by $v_p \in \mathbb{R}^3$. Affine velocity $C_p \in \mathbb{R}^{3\times 3}$ is introduced to capture the non-linear local deformations within the object. A deformation field $\varphi_p : \mathbb{R}^3 \to \mathbb{R}^3$ is used to record the initial and final positions of each particle in each time step. The deformation gradient $F_p \in \mathbb{R}^{3\times 3}$ represents the extent of deformation relative to its initial state, which can be derived as:

$$F_p = \frac{\partial \varphi_p}{\partial x_p}(x_p) \tag{1}$$

4.1.2 Particle-to-Grid.

This process maps particle properties to grid nodes through interpolation, using quadratic B-spline weighting to compute the influence of each particle on the surrounding grid nodes. The mass and the momentum of the particles are then accumulated onto the corresponding grid nodes based on these weights. The mass M_i of the i-th grid node is:

$$M_i = \sum_{j \in \mathbb{G}_i} \sum_{p \in \mathbb{P}_j} w_{jp} m_p \tag{2}$$

where $\mathbb{G}_i$ denote the $3 \times 3 \times 3$ grid nodes containing the i-th grid node and its neighboring grid nodes, $\mathbb{P}_j$ denote the particles inside the j-th grid, and w_{ij} denote the weight parameter between the i-th grid node and the j-th particle, calculated using quadratic B-Spline interpolation, and m_p denote the mass of the p-th particle.

The momentum MG_i of the i-th grid node can be obtained by calculating the momentum resulted

from the particle motion MM_i and the momentum resulted from the elasticity ME_i:

$$MG_i = MM_i + ME_i \tag{3}$$

MM_i is calculated by collecting the velocity and affine velocity of the nearby particles (A temporal superscript is utilised to distinguish the parameters at each different time step, e.g., $v_p^{(k)}$ denoting the velocity at time step k):

$$MM_i = \sum_{j \in \mathbb{G}_i} \sum_{p \in \mathbb{P}_j} w_{jp} \left(m_p v_p^{(k)} + C_p^{(k)} (X_j - x_p^{(k)}) \right) \tag{4}$$

where X_j denotes the position of the j-th neighboring node of the i-th grid node.

ME_i is obtained by accumulating the elastic stress from neighbouring particles:

$$ME_i = -\Delta t \sum_{j \in \mathbb{G}_i} \sum_{p \in \mathbb{P}_j} \frac{4}{\Delta X^2} w_{jp} V_p^0 S_p^{(k)} (X_j - x_p^{(k)}) \tag{5}$$

where Δt is the time interval between two adjacent steps, ΔX is the grid node interval, V_p^0 denotes the initial particle volume, S_p is the elasticity force for the p-th particle.

4.1.3 Grid Operation.

The velocity on the i-th grid node can be obtained given the grid node momentum and the grid node mass:

$$V_i = \frac{MG_i}{M_i} \tag{6}$$

Note that the grid node velocity is only for later parameter updates, and the position of the grid will not change in the simulation.

4.1.4 Grid-to-Particle.

The states of the particles are then updated with the previous states of the grid nodes and the particles. The velocity $v_p^{(k+1)}$, the affine velocity $C_p^{(k+1)}$ and deformation gradient $F_p^{(k+1)}$ at time step $k+1$ can be obtained by:

$$v_p^{(k+1)} = \sum_{i \in \mathbb{G}'_p} w_{ip} V_i^{(k)} \tag{7}$$

$$C_p^{(k+1)} = \frac{\Delta X^2}{\Delta t} \sum_{i \in \mathbb{G}'_p} w_{ip} v_p^{(k+1)} \frac{X_i - x_p^{(k)}}{\Delta X} \tag{8}$$

$$F_p^{(k+1)} = \left(I + \Delta t C_p^{(k+1)} \right) F_p^{(k)} \tag{9}$$

where G'_i represents the $3 \times 3 \times 3$ grid nodes surrounding the p-th particle.

4.1.5 Particle Operation.

We will apply specific boundary conditions to constrain the motion of certain particles. Particles within the rigid body $\mathbb{R}$ will share the same velocity v (e.g., the rigid indenter and sensor actuator), while those in the fixed region $\mathbb{B}$ will have a velocity of 0 (e.g., the inner support layer of the sensor membrane if the sensor is fixed):

$$v_p^{(k+1)} = \begin{cases} v & \text{if } p \in \mathbb{I}, \\ 0 & \text{if } p \in \mathbb{B}, \end{cases} \tag{10}$$

Finally, the position of each particle $x_p^{(k+1)}$ at $k+1$ time step can be calculated by:

$$x_p^{(k+1)} = x_p^{(k)} + \Delta t \, v_p^{(k+1)} \tag{11}$$

The entire flow chart of the MPM iteration is sketched in Algorithm 1.

Algorithm 1 Material Point Method Iteration

```
Input: Particles of the sensor skin and the objects (P); Number of the grid nodes (n); Material
parameters of the skin (E and ν); Relative velocity of the contact v_r; Index of interested particles
(boundary conditions, contact surfaces, actuator surfaces).
Output: Positions of the deformed particles.
Discrete the model into particles using the free mesh algorithm.
Initialise the values of x_p^(0), F_p^(0), C_p^(0), and v_p^(0).
Divide the simulation space into grids.
while not terminal do
    for each grid i do
        Collect the mass M_i and momentum MG_i of grid i by Eq. (10-13).
        Update the grid velocity V_i by Eq. (14).
    end for
    for each particle p do
        Update parameters v_p^(k+1), F_p^(k+1), and C_p^(k+1) by Eq. (15-17).   ▷ k represents the k-th
time step
    end for
    Boundary conditions applied by Eq. (18).
    Particle position updated by Eq. (19).
    Terminal check: whether the target pose of the indenter/sensor is achieved.
end while
```

4.2 Data collection setup

We mainly collected tactile data from the finger-shaped GelTip sensor [2] and the cubic-shaped GelSight sensor [3]. The GelTip dataset was used to evaluate the accuracy of the simulator and validate Sim2Real tasks such as contact object classification and slip detection, while the GelSight dataset was solely utilised for contact safety assessment.

4.2.1 Data collection on GelTip sensor.

We set up a data collection platform as shown in Fig. 4a. The GelTip sensor is securely mounted on the table, while the indenter and the ATI NANO 17 force sensor are attached to the UR5e robot arm for different contact positions and orientations. The sensor has multiple slots in its body and can be rotated in 90-degree increments for flexible positioning by selecting different slots to connect to the table base. We also replicate the same sensor and indenter configuration in both the proposed simulator and the FEM, collecting data in both environments for comparison. A total of 14 distinct indenter shapes were utilised, with 10 shapes in the seen group for the STN model training and 4 in the unseen group for testing.

The data acquisition process is shown in Fig. 4d. The contact path extends from the tip to the base of the membrane, with contact points distributed at 90-degree intervals around the circumference to ensure full coverage of the sensor surface. In each acquisition cycle, the robotic arm first positions the indenter at each contact point, maintaining a perpendicular orientation to the contact surface, and then performs a sequence of actions: normal contact, shear motion, and normal release. During both the normal contact and shear motion phases, tactile images and force data are recorded synchronously at a frequency of 30 Hz. The pseudocode for the data acquisition process is provided in Algorithm 2.

The data collection parameters are defined as follows and are illustrated in Fig. 4d: the indenter rotates within the tip region from $0°$ to $90°$ with an interval of $\Delta\alpha = 10°$, and moves within the base region from 0 mm to 12 mm with a step size of $\Delta d = 1$ mm. Four contact paths are evenly distributed around the sensor circumference at $90°$ intervals. At each contact position, the indentation depth z ranges from 0 mm to 1 mm with a depth increment of $\Delta z = 0.1$ mm. For each indentation depth, shear motion is applied in 8 different directions, with an angular interval of $\Delta\beta = 45°$. The shear displacement along the x or y axis varies from 0 mm to 0.5 mm, with a step size of $\Delta x = 0.1$ mm and $\Delta y = 0.1$ mm. The data collection setup is shared across the real world, SimTac, and FEM simulation environments.

4.2.2 Data collection on GelSight sensor.

The data collection platform for the GelSight sensor, shown in Supplementary Fig. 1a, closely resembles the setup used for the GelTip sensor. We used the same indenters as the GelTip sensor and replicated the setup in both the proposed simulator and the FEM environment, as shown in Supplementary Fig. 1b. The data collection process is illustrated in Supplementary Fig. 1c and in Algorithm 3, where the contact points are distributed in a square grid pattern, centred around the sensor surface, ranging from -3 mm to 3 mm, with a spacing of $\Delta d = 1.5$ mm. The contact depth z varies from 0 to 1.2 mm, with a depth interval of $\Delta z = 0.1$ mm. The indenter applies only normal contact, without any shear motion. All other settings remain consistent with those used in the data collection for the GelTip sensor.

Algorithm 2 Data Collection on GelTip Sensor

Input: Surface contact points $P = \{P_1, ..., P_m\}$, where $P_i = \{p_1, ..., p_n\}$ contains all contact points in depth, $p_j = \{x, y, z\}$ denotes the coordinate of a contact point, 8 shear directions in each p_j, $S = \{s_1, ..., s_8\}$

procedure MoveToContactPoints(P, m)
    for $i = 0 \ldots m$ **do**
        $p_0 \leftarrow P_i[0]$ ▷ origin point p_0 without contact
        $moveTo(p_0)$
        $n_{p0} \leftarrow \frac{p_0}{\|p_0\|}$ ▷ calculate normal vector of contact point p_0
        $adjustOrientation(n_{p0})$ ▷ make the indenter perpendicular to the sensor surface
        for $j = 1 \ldots n$ **do**
            $p_1 \leftarrow P_{ij}$
            $moveTo(p_1)$, $record\_image\_and\_force()$ ▷ Normal increases
            for $k = 1 \ldots 8$ **do**
                $moveTo(s_k)$, $record\_image\_and\_force()$ ▷ Shear motion
                $moveTo(p_0)$ ▷ Normal decreases
            end for
        end for
    end for
end procedure

Algorithm 3 Data Collection on the GeSight Sensor

Input: Surface contact points $P = \{P_1, ..., P_m\}$, where $P_i = \{p_1, ..., p_n\}$ contains all contact points in depth, $p_i = \{x, y, z\}$ denotes the coordinate of a contact point.

procedure MoveToContactPoints(P, m)
    for $i = 0 \ldots m$ **do**
        $p_0 \leftarrow P_i[0]$ ▷ origin point p_0 without contact
        $moveTo(p_0)$
        for $j = 1 \ldots n$ **do**
            $p_1 \leftarrow (p_0[0], p_0[1], P_{ij}[2])$
            $moveTo(p_1)$, $record\_image\_and\_force()$ ▷ Normal increases
            $moveTo(p_0)$, $record\_image\_and\_force()$ ▷ Normal decreases
        end for
    end for
end procedure

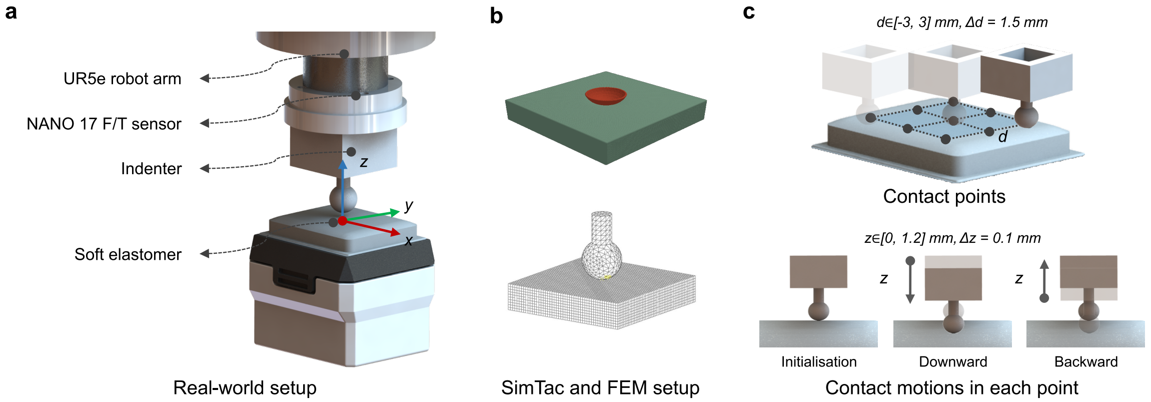

Figure 12: Data collection setup on the GelSight sensor. **a-b**, Real-world, SimTac and FEM data collection platform for the GelSight sensor. **c**, The movement trajectory of the indenter during the GelSight data collection process.

4.3 The elephant-trunk-shaped sensor

4.3.1 Simulated sensor setup.

We first designed and implemented a sensor prototype within the simulation environment, as illustrated in Fig. 9b. The surfaces of the two protrusions at the sensor tip were designated as actuator surfaces, where specific particle velocities were applied to achieve opening and closing motions. The outer surface of the elastomer was assigned as the reflective layer, whose deformations were leveraged for optical rendering to generate tactile images. The inner surface was defined as the support layer, with particle velocities constrained to zero. A 120-degree wide-angle camera was positioned at the base of the membrane to capture deformations occurring within the tip region. A ring of red, white, blue, and green LEDs was arranged around the membrane base to provide structured internal illumination.

The simulation pipeline is outlined in Fig. 9c. We first employed the Unstructured Mesh Algorithm [4] to generate a $C3D4$ mesh (four-node tetrahedral elements) with a particle spacing of $h = 0.4$ mm to discretise the sensor membrane. The nodal coordinates of the generated mesh were extracted to initialise the positions of the membrane particles. The object in contact with the sensor was modelled as a rigid body and discretised via uniform sampling using *Open3D* [5] into 1×10^6 particles, with all particles sharing the same velocity. The material properties of the membrane were defined by a Young's modulus of $E = 1.45 \times 10^5$ kPa and a Poisson's ratio of $v = 0.45$. The simulation environment was configured with 256 grid nodes over a total grid length of 70 mm. After initialisation, linear and non-linear light fields were generated offline according to the geometric model of the sensor membrane. Optical rendering was subsequently performed using the depth maps obtained from deformation simulations. The ambient background images and the foreground images generated by diffuse and specular reflections were composited to synthesise the final tactile images.

4.3.2 Real sensor setup.

The real sensor consists of four key components: the actuator module, the optical module, the skin module and the lighting module, as shown in Fig. 9d. The overall dimensions of the sensor are 34 mm × 26 mm × 50 mm. The actuator module comprises two sliders, which are driven by an external force to enable opening and closing movements. The optical module includes a 5MP-OV5640-USB camera, capable of capturing images at a 1920 × 1080 resolution with a 30 Hz frame rate. The camera is equipped with a 120-degree distortion-free fixed-focus lens with a 1.5 mm focal length. The skin module consists of three layers: a transparent rigid support layer, a transparent elastomer layer, and a reflective coating layer.

The support layer provides structural reinforcement and limits deformation, the elastomer layer enables deformation while facilitating light propagation, and the reflective coating blocks external light while enhancing internal reflection. The rigid support layer is fabricated from a transparent resin using a Formlabs Form-3 3D printer and polished to improve transparency. The elastomer layer is cast using a 3D-printed mould, with the mould surface polished for better clarity. The elastomer is made of silicone (XP-565 from Silicones, Inc.), mixed at a 1:15 ratio. The reflective coating layer consists of a membrane mixed with aluminium powder and reflective pigment. The lighting module consists of an LED ring, which serves as the internal light source. We designed a programmable ring-shaped LED system (WS2812 control chip, 1615 SMD LEDs) that allows precise control over the light sources through adjustable RGB values and intensity settings, ensuring accurate calibration with the simulated lighting conditions. The LEDs are arranged in a red-white-blue-green sequence, with equal brightness levels of 1:1:1:1. The fabrication process is detailed in Fig. 9e.

4.4 Sim2Real task setup

4.4.1 Contact object classification setup.

We used the ResNet50 [6] architecture as our machine learning model, which uses tactile images as input and outputs the probability for each shape class. Following the data collection method described in Supplementary 4.2, only vertical contact was applied, without any shear motion. A total of 8,016 tactile image pairs were collected from the contact of different-shaped indenters with the GelTip sensor in both real and simulated environments. These image pairs were divided into training, validation, and test sets in a ratio of 3:1:1. During the training process, all parameters of the convolutional layer are initialised using a pre-trained model on ImageNet [7] to enhance feature extraction capabilities. The classification model was trained with a batch size of 32 for 30 epochs using the Adam optimiser with a learning rate of 0.001 to minimise the mean squared error (L1 loss).

4.4.2 Slip detection setup.

We propose a deep neural network (DNN)-based approach for slip detection, which takes a sequence of eight tactile images as input and outputs a binary classification indicating whether slip has occurred or not. Following the data collection method described in Supplementary 4.2, we collected

a total of 832 tactile sequences involving shear contact between various indenter shapes and the GelTip sensor, in both simulated and real-world settings. The contact area was restricted to the sidewall region, which is the primary contact area when the sensor grasps an object. The dataset is balanced, with an equal number of slip and non-slip samples (1:1 ratio). To accurately label the data, we first replicated the real-world experimental setup and shear loading process in FEM, enabling the recording of tangential forces applied to the sensor. The onset of slip is defined as the point where the tangential force curve transitions from increasing to decreasing. The displacement at this turning point is used as the slip detection threshold: sequences with displacements below this threshold are labelled as non-slip, while those above are labelled as slip. This thresholding method is applied to all simulated sequences. For the real-world data, we mounted a force sensor on the indenter to directly measure tangential force, using the same criterion for consistent labelling.

The model is trained exclusively on simulated data, with 60% used for training, 20% for validation, and 20% for testing. All real-world data is reserved for testing to assess sim-to-real transfer performance. The network architecture consists of a convolutional neural network (CNN) for spatial feature extraction and a recurrent neural network (RNN) for temporal decision-making. Specifically, we use a VGG-19 backbone [8] pre-trained on ImageNet [7] to encode tactile features, followed by a long short-term memory (LSTM) network [9] to model temporal dependencies across frames. The model is trained using the Adam optimiser with a learning rate of 0.0005, a batch size of 4, and cross-entropy loss, for 15 epochs.

4.4.3 Contact safety assessment setup.

We constructed a regression model using the ResNet50 architecture [6], which takes tactile images as input and predicts the safety coefficient, a metric used to quantify the magnitude of local contact pressure. As described in Supplementary 4.2, we collected 2,548 tactile images in SimTac, which were obtained from the contact between different-shaped indents and a GelSight sensor. The image data were split into training, validation, and test sets with a 3:1:1 ratio. Additionally, 559 tactile images were collected from real-world scenarios for Sim2Real transfer evaluation. For each simulated image, we recorded the local peak distributed force in the predicted force field, normalised it to the range of 0 to 1, and used it as the label representing the contact safety coefficient. In the real-world scenario, the tactile image acquisition conditions were identical to those in SimTac, and the aligned data shared the same safety coefficient labels. The regression model was trained with a batch size of 32 for 50 epochs, using the Adam optimiser with a learning rate of 0.001, aiming to minimise the MAE.

4.5 FEM model for generating ground truth data

We first model the flexible membrane of the sensor along with the objects it contacts. The membrane is divided into several regions: the outer surface, the inner surface, and the active deformation surface. The outer surface is the area that interacts with the environment and where the primary deformations occur. The inner surface connects to the internal rigid structure and serves as the boundary condition region. The active deformation surface is relevant for membranes capable of

active motion, such as those used in translational or rolling sensors.

We then define the material properties for the simulation as follows: contacting objects are modelled as linear elastic materials, characterised by Young's modulus E and Poisson's ratio v. The sensor membrane is modelled as a superelastic material using the Neo-Hookean formulation, with parameters $C_{10} = \frac{\mu}{2}$ and $D_1 = \frac{2}{\lambda}$, where μ and λ denote the shear modulus and the first Lamé parameter, respectively. Under small strains and rotations, the Neo-Hookean model reduces to linear elasticity, with μ and λ related to E and v by:

$$\mu = \frac{E}{2(1+\nu)}, \quad \lambda = \frac{E}{3(1-2\nu)} \tag{12}$$

Finally, we configure the contact and loading conditions. The normal contact between the membrane and the object is modelled as hard contact, utilising a normal-Lagrange contact formulation to mitigate penetration effects. In the tangential direction, a penalty-based friction contact model is employed, allowing for elastic sliding when the object sticks to the sensor. A friction coefficient μ_0 is defined to regulate the roughness of the contact interface surface. For the rigidly supported areas of the membrane, boundary conditions are constrained to limit displacement. Loads are applied to the objects or the actively deforming regions of the membrane, the surfaces are driven to move in a specified direction by defining the direction of motion and velocity. Details on the FEM parameters setup can be found in Algorithm 4.

4.6 Comparison with existing methods

4.6.1 STN vs PointNet++: deformation and force prediction

PointNet++ [10] is a hierarchical point cloud feature extraction network that can capture both local and global features from point clouds, enabling the prediction of continuous values for individual points or the entire point cloud. This makes it particularly well-suited for point cloud regression tasks, allowing for stable and accurate MPM-to-FEM mapping. We trained both STN and PointNet++ on the same dataset and evaluated them across all datasets. The comparison results are shown in Table 1.

Table 1: Comparison of prediction accuracy and efficiency between STN and PointNet++.

**Response**	**Model**	**MAE-X**	**MAE-Y**	**MAE-Z**	**MAE**	**FPS**
Deformation (mm, $\times 10^{-4}$)	PointNet++	12	15	14	14	4.5
	STN(ours)	2.2	3.9	2.3	2.8	76
Force (N, $\times 10^{-6}$)	PointNet++	134	35	25	65	4.5
	STN(ours)	10	5.1	6.7	7.4	76

As shown in the table, STN achieves significantly lower prediction errors than PointNet++ in both deformation and force field estimation, while also running approximately 17 times faster. This advantage stems from STN's ability to perform convolution directly on sparse tensors, preserving the spatial structure of the particle cloud and enabling more stable and accurate prediction of continuous values. Although PointNet++ can capture local features, it relies on point sampling and feature

Algorithm 4 FEM Setup for GelTip Sensor

Create File
  File→New Model Dataset→With Standard/Explicit Model
Part
  File→Import→Part→Import GelTip membrane and indenter
  Tools→Reference Point→Select the indenter center point
Property
  if Sensor Membrane
    Set Hyperelastic Material
      Density→$Mass\ Density = 0.001$
      Hyperelastic→Neo Hooke→Coefficients→$C10 = 0.025, D1 = 4.1379$
  else if Indenter
    Set elastic Material
      Density→$Mass\ Density = 0.008$
      Elastic→$Young's\ Modulus = 210, Poisson's\ Ratio = 0.3$
Assembly
  Create instance: Import membrane and indenter→Set initial pose
  Create set: Boundary Surface→inner support layer, Contact Surface→outer coating layer, Reference Point→indenter centre point
Step
  Create Step: Static, General→$Timeperiol = 1$→turn on Nlgeom
  Create Field Output: $Domain = Contact\ Surface$→$Frequency = 0.025s$→$Ouput = UT, NFORC$
Interaction
  Create Interaction Property:
    $Normal\ Behavior =$ "$HardContact$"
    $Tangential\ Behavior = Penalty, FrictionCoeff = 0.3$
  if Indenter
    Create Constraint→Coupling
Load
  Create Boundary Condition:
    Fix→Sensor Boundary Surface→Type=Symmetry/Antisymmetry/Encastre
    Set Load→Indenter→Type=Displacement→Normal/Shear, interval=0.1 mm
Mesh
  if Indenter
    $Approximate\ Global\ Size = 0.8$→$Element\ Shape = Tet$
  else if Sensor Membrane
    $Approximate\ Global\ Size = 0.4$→$Element\ Shape = Hex(Structured)$
Job
  Generate ODB files
Post-Processing
  Run script to extract the displacement/force field from ODB file
End Algorithm

Table 2: Quantitative comparison of optical response with advanced tactile simulators.

	SSIM↑	MAE↓ ($\times 10^{-2}$)	MSE↓ ($\times 10^{-4}$)	PSNR↑
Ours	0.92	1.8	6.5	80
Gomes et al. [11]	0.89	2.0	9.2	76
TACTO [12]	0.85	2.3	10.5	71

aggregation, which may limit its ability to capture fine-grained local variations. In addition, STN's sparse representation avoids computation on empty or redundant points, reducing memory usage and computational cost, whereas PointNet++ requires feature computation for each local neighborhood, causing the computational overhead to increase substantially as the point cloud density grows.

We further visualized the predictions generated by STN and PointNet++, as illustrated in Supplementary Fig. 9. For the predicted deformation maps, STN produces more continuous results with smoother transitions compared to PointNet++. Additionally, STN demonstrates higher accuracy in predicting force maps, particularly for tangential forces.

4.6.2 Optical response comparison

For optical response simulation, a simulator that supports tactile sensors with curved morphologies must model the deformation of the curved membrane, the distortion of its projection onto the camera plane, and the propagation of light within the curved membrane. Therefore, we compared SimTac with representative open-source simulators that support tactile sensors with curved morphologies [11, 12]. Both [11] and [12] employ a model penetration approach to capture membrane deformation, while also allowing for the simulation of light propagation within the membrane.

As shown in Supplementary Fig. 10, SimTac produces more realistic contact deformation patterns and color distributions than the baselines. This improvement mainly benefits from our physics-based contact deformation model and the linear/nonlinear light-field rendering method. In addition, we conducted quantitative evaluations using standardized metrics, as summarized in Table 2, where SimTac achieves the best overall performance.

For mechanical response simulation, we evaluated existing open-source simulators capable of curved tactile sensor modeling and conducted a direct comparison with [13], which also employs an FEM-based approach. The hemispherical sensor model in [13] was adapted to a finger-shaped sensor, and contact forces were measured under various indenter shapes. As shown in Table 3, SimTac provides more accurate force predictions when compared with real-world measurements. This improvement can be attributed to SimTac's higher mesh resolution (5K, 70 FPS), whereas [13] suffers from a substantial drop in efficiency at higher mesh densities (5K, 4 FPS), despite its lower-resolution performance (0.5K, 30 FPS).

4.7 Adapting STN to novel sensor geometries via fine-tuning

To evaluate the generalization capability of the STN model to previously unseen sensor geometries, we conduct transfer learning experiments on an elephant-trunk-shaped tactile sensor. This sen-

Table 3: Quantitative comparison of mechanical response with tactile simulator [13].

	MAE–X	MAE–Y	MAE–Z	MAE–All
[13]	12.5	13.6	18.3	14.8
Ours	6.3	9.2	13.2	10.3

sor geometry differs fundamentally from the GelTip sensors used during pre-training, providing a challenging test case for cross-geometry adaptation.

For this new sensor geometry, the transfer process relies solely on a limited set of simulation-generated deformation-force pairs relevant to the new geometry (MPM-FEM data), as the pre-trained model has already captured the fundamental physical relationship between deformation and contact forces. The STN model pre-trained on GelTip sensors is subsequently adapted to the new, more complex biomorphic sensor via transfer learning, facilitating efficient knowledge transfer to previously unseen sensor morphologies and supporting expedited sensor prototyping through simulation. In this study, the dataset comprises 282 contact scenarios collected over approximately 6 hours, with 80% allocated for training and 20% for testing, and no network layers were frozen during training.

Supplementary Fig. 11a visualizes the predicted deformation and force fields, showing strong particle-level consistency with the ground truth. Supplementary Fig. 11c presents the distributions of prediction errors for both deformation and force under contacts with different indenters. Quantitatively, the fine-tuned model achieves high prediction accuracy on the new sensor geometry. The MAEs of the deformation maps along the X, Y, and Z directions are X, Y, and Z directions are 5.83×10^{-4} mm, 5.72×10^{-4} mm, and 3.55×10^{-4} mm, respectively, while the MAEs of the force maps along X, Y, and Z are 8.3×10^{-6} N, 12.9×10^{-6} N, and 6×10^{-6} N. In addition, the total force predicted by the fine-tuned STN closely matches real-world measurements, with percentage errors of 15.5%, 16.1%, and 12.1% along the X, Y, and Z axes.

As shown in this experiment, the fine-tuning process substantially reduces both data collection and training costs, i.e., only a limited amount of data (approximately 15% of the pre-training data) is needed, which is 85% reduction compared to training a model from scratch. The result demonstrates the practical efficiency of the proposed transfer learning approach.

To further improve the adaptability of the model to new, complex sensor geometries, we plan to explore meta-learning strategies in future work. By treating each sensor's geometry as a conditioning input and learning shared initialization parameters, the model can be rapidly fine-tuned with only a few samples. Using point cloud representations to extract geometry-aware features enables cross-geometry feature sharing, allowing the model to quickly converge on new designs while reducing reliance on large-scale data. This approach aims to reduce reliance on FEM data and enable rapid convergence when facing previously unseen shapes, thereby accelerating simulation and prototyping of novel sensor designs.

4.8 Simulation of fine surface textures

To evaluate SimTac's capability in simulating fine surface textures, we design two objects with distinct microscopic surface patterns. Each object measures $1.2 \times 1.0 \times 0.3$ cm and contains texture features in the sub-millimeter scale, as shown in Supplementary Fig. 12. As these fine-grained features exceed the resolution limits of conventional 3D printing, all experiments are conducted in a fully simulated environment.

Tactile images of a GelTip sensor in contact with the two objects are generated using the proposed optical rendering strategy. Three particle resolutions are evaluated: 25K, 250K, and 1.2M particles. As shown in Supplementary Fig. 12, increasing the particle resolution enables progressively more accurate reconstruction of the objects' microscopic surface textures. Among the evaluated settings, 250K particles provide a favorable trade-off between texture reconstruction fidelity and computational efficiency. Moreover, due to the surface tension and elastic rebound properties of silicone materials, the gel surface does not perfectly conform to every minute surface feature during contact. The proposed particle-based deformation simulation faithfully reproduces this physical behavior, demonstrating the robustness of the rendering model under micro-texture conditions.

This rendering strategy is a local illumination model that primarily accounts for direct lighting from light sources, deliberately omitting multiple light bounces and indirect illumination to maintain computational efficiency. As a result, specular surfaces under large deformations may exhibit discontinuities or less realistic appearances due to the absence of indirect lighting and environmental reflections. The model allows for partial representation of variations in material or texture reflectivity by adjusting the reflection coefficients, though its accuracy can be limited when simulating materials with more complex reflective properties, such as metals used in the sensor coating layer.

4.9 Zero-shot Sim2Real slip detection under dynamic interactions

To rigorously evaluate the zero-shot Sim-to-Real slip-detection performance of the trained model, we conduct a set of dynamic and uncontrolled real-world experiments, including water-pouring and object-striking tasks, as shown in Supplementary Fig. 13.

In the water-pouring experiment, two cups of different sizes are firmly grasped by the gripper, after which water is continuously poured into the cups, generating a gradually increasing and highly dynamic load on the tactile fingers. In the object-striking experiment, four deformable objects with varying stiffness properties are examined, and dynamic impulsive loads are induced by striking the grasped objects with a hammer. Across both experimental scenarios, the tactile fingers are required to predict slip under continuously varying force conditions. Furthermore, two distinct sensor illumination configurations are evaluated. For each object, 20 slip trials and 20 non-slip trials are conducted, and the slip-detection success rate is subsequently recorded.

As summarized in Table 4, the model achieved 100% slip prediction and 95% non-slip prediction on Cup1, and 95% slip prediction and 100% non-slip prediction on Cup2 in the water-pouring experiment. In the object-striking experiment, objects with well-defined geometric features (cron toy and toothpaste) achieved slip prediction accuracies of 100% and non-slip prediction accuracies of 90% and 95%, respectively. In contrast, performance decreased to 40% and 20% for extremely

soft objects, including the Jellycat toy and sponge. This performance drop can be attributed to their low stiffness, which limits the tactile sensor's ability to capture sufficiently distinctive surface geometry within the contact region, thereby making reliable slip detection more challenging. We further evaluated different sensor illumination configurations by adjusting the brightness of the sensor LEDs in various colors. As reported in Table 4, slip detection achieved comparable success rates (varied by up to 5%) under different sensor lighting conditions, indicating that the model trained in simulation exhibits strong robustness and stability when transferred to real sensors with variations in illumination.

Table 4: Success rates of slip detection in water pouring and object striking task.

Task	Object	Sensor Lighting 1		Sensor Lighting 2	
		Slip	Non-Slip	Slip	Non-Slip
Water Pouring	Cup 1	100%	95%	100%	90%
	Cup 2	95%	100%	90%	95%
Object Striking	Soft Cron Toy	100%	90%	100%	95%
	Toothpaste	100%	95%	95%	90%
	Jellycat Toy	40%	90%	30%	85%
	Sponge	20%	85%	20%	80%

These results highlight the strong zero-shot Sim-to-Real generalization capability of the proposed approach under dynamic interactions, while also indicating potential limitations when handling highly compliant objects. In future work, we plan to expand the training dataset to include a broader range of deformable objects and richer surface texture patterns, as well as a wider variety of dynamic contact scenarios beyond primarily quasi-static interactions, to further improve the robustness and stability of the prediction model.

4.10 Trade-off between computational efficiency and accuracy

We first evaluated the computational performance of SimTac using four sensors with distinct geometric complexities, under varying particle densities and rendering resolutions, as summarized in Table 5. The results indicate that sensors with different shapes exhibit comparable iteration frame rates for deformation when the particle count is the same. This can be attributed to the nature of the MPM, which performs iterations by transferring momentum between particles and the surrounding virtual grid. Consequently, the computational cost is primarily determined by the number of particles rather than the sensor geometry. In contrast, the simulation frame rate of the optical response module decreases as the number of image pixels increases but remains independent of particle count and sensor geometry, since the light-field rendering computes RGB values per pixel rather than per particle. For the mechanical response simulation, the frame rate is positively correlated with the particle count and remains unaffected by the particle cloud shape, as the STN network voxelizes the particle cloud and performs convolutions on the voxel features. Overall, these results demonstrate that the computational efficiency of the simulation system is largely independent of sensor geometry, highlighting its applicability to sensors of diverse and complex shapes.

Table 5: Computational performance evaluation across sensors with different geometries.

	Deformation Iteration FPS			Optical Response FPS			Mechanical Response FPS		
Particles	40K	300K	1.3M	320×240	640×480	1280×960	1K	5K	25K
Finger	250	33.2	9.8	100	25	10	100	76	62
CatPaw	252	32.9	9.8	100	25	10	104	70	60
Elephant	253	33.5	10.0	100	25	10	101	75	62
Octopus	249	33.7	9.6	100	25	10	98	69	60

Table 6: Accuracy evaluation with different particle densities.

	Optical Accuracy (SSIM↑)			Mechanical Accuracy (Total Force Error↓, %)		
Particles	40K	300K	1.3M	1K	5K	25K
Finger	0.87	0.91	0.92	25.0%	10.3%	8.5%
Elephant	0.85	0.89	0.90	29.5%	14.6%	12.3%

We then evaluated the simulator accuracy with varying particle densities using finger-shaped and elephant-trunk-shaped sensors, for which corresponding physical sensors were fabricated. As shown in Table 6, the SSIM between rendered and real images increases with particle density, reaching a balance between accuracy and computational efficiency at approximately 300K particles. Similarly, the accuracy of mechanical predictions improves with particle count, achieving an optimal trade-off at around 5K particles. These observations indicate that both optical and mechanical responses become more accurate as particle density increases, although the rate of improvement gradually diminishes, allowing a practical trade-off between accuracy and computational cost.

4.11 Multiple contact simulation

We assessed the SimTac's capability for simulating sensors with multiple, simultaneous contact points, as visualized in Supplementary Fig. 14. Experiments with different two-point contact combinations and three-point contacts demonstrate that the trained model can effectively simulate multi-contact scenarios. The MAEs of the deformation maps along the X, Y, and Z directions are 4.42×10^{-4} mm, 4.71×10^{-4} mm, and 4.24×10^{-4} mm, respectively, while the MAEs of the force maps along X, Y, and Z are 1.14×10^{-5} N, 1.34×10^{-5} N, and 1.11×10^{-4} N. As with all learning-based predictive models, additional training data can further enhance the prediction accuracy.

References

1. Chen Z, Zhang S, Luo S, Sun F, and Fang B. Tacchi: A pluggable and low computational cost elastomer deformation simulator for optical tactile sensors. IEEE Robotics and Automation Letters 2023;8:1239–46.

2. Gomes DF, Lin Z, and Luo S. GelTip: A finger-shaped optical tactile sensor for robotic manipulation. In: *2020 IEEE/RSJ International Conference on Intelligent Robots and Systems (IROS)*. 2020:9903–9.
3. Yuan W, Dong S, and Adelson EH. Gelsight: High-resolution robot tactile sensors for estimating geometry and force. Sensors 2017;17:2762.
4. Bern MW and Plassmann PE. Mesh generation. Handbook of computational geometry 2000;38.
5. Zhou QY, Park J, and Koltun V. Open3D: A modern library for 3D data processing. arXiv preprint arXiv:1801.09847 2018.
6. Koonce B and Koonce B. ResNet 50. Convolutional neural networks with swift for tensorflow: image recognition and dataset categorization 2021:63–72.
7. Deng J, Dong W, Socher R, Li LJ, Li K, and Fei-Fei L. Imagenet: A large-scale hierarchical image database. In: *2009 IEEE conference on computer vision and pattern recognition*. 2009:248–55.
8. Tammina S. Transfer learning using vgg-16 with deep convolutional neural network for classifying images. International Journal of Scientific and Research Publications (IJSRP) 2019;9:143–50.
9. Graves A and Graves A. Long short-term memory. Supervised sequence labelling with recurrent neural networks 2012:37–45.
10. Qi CR, Yi L, Su H, and Guibas LJ. Pointnet++: Deep hierarchical feature learning on point sets in a metric space. Advances in neural information processing systems 2017;30.
11. Gomes DF, Paoletti P, and Luo S. Beyond flat gelsight sensors: Simulation of optical tactile sensors of complex morphologies for sim2real learning. Robotics: Science and Systems 2023.
12. Wang S, Lambeta M, Chou PW, and Calandra R. Tacto: A fast, flexible, and open-source simulator for high-resolution vision-based tactile sensors. IEEE Robotics and Automation Letters 2022;7:3930–7.
13. Si Z, Zhang G, Ben Q, et al. DIFFTACTILE: A Physics-based Differentiable Tactile Simulator for Contact-rich Robotic Manipulation. The International Conference on Learning Representations 2024.
14. Lepora NF, Lin Y, Money-Coomes B, and Lloyd J. Digitac: A digit-tactip hybrid tactile sensor for comparing low-cost high-resolution robot touch. IEEE Robotics and Automation Letters 2022;7:9382–8.

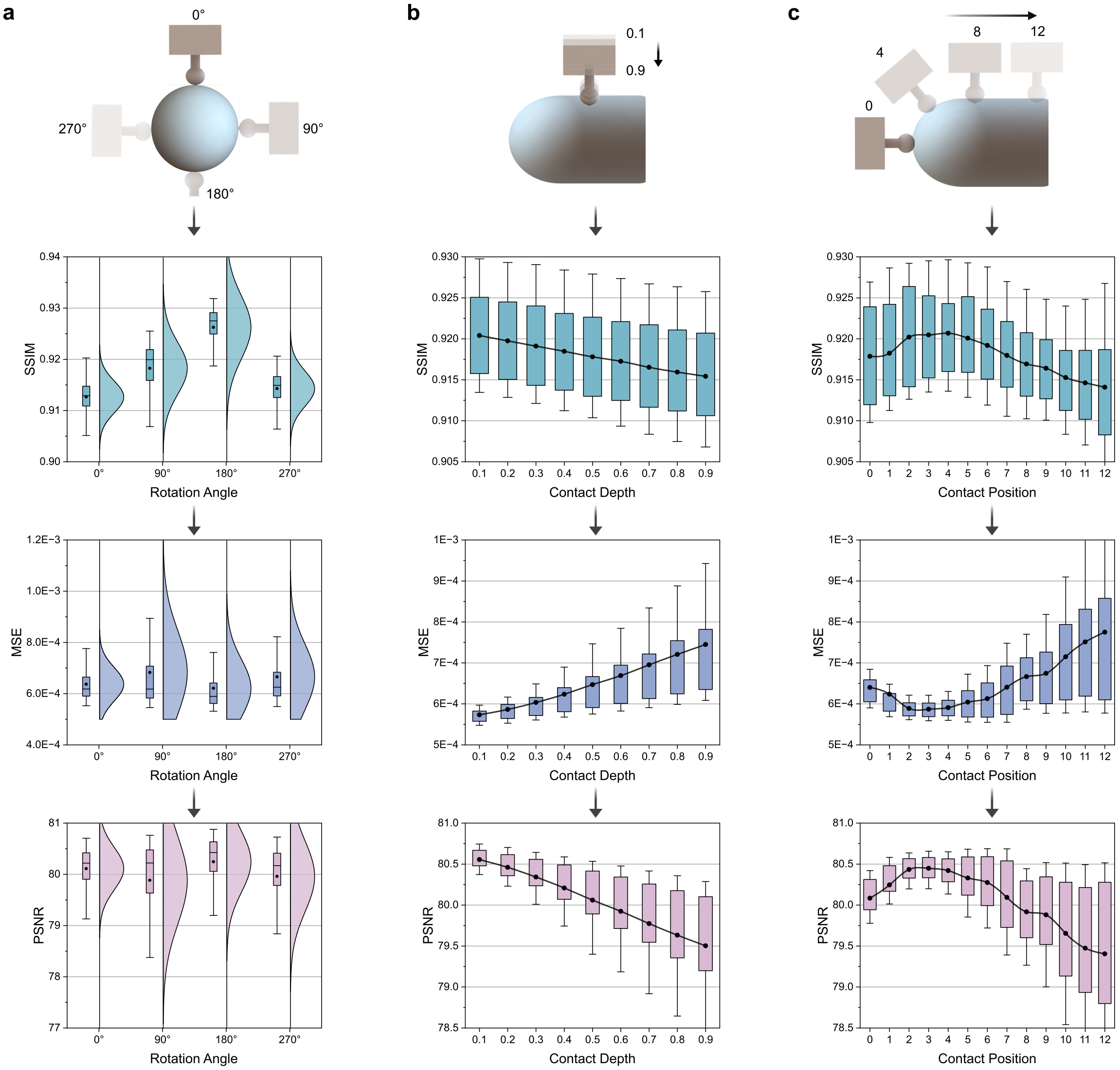

Figure 13: Evaluation of optical response simulation under different contact scenarios. The figure presents different contact scenarios (**a**, different contact angles around the sensor; **b**, different contact depths; **c**, different contact positions) and corresponding metrics analysis using SSIM, MSE, and PSNR. The similarity between simulated and real images exhibits slight fluctuations at different contact angles and decreases with increasing compression depth. This trend arises from variations in RGB light distribution across contact positions and the expansion of the deformation area at higher compression depths, which leads to greater rendering errors. Additionally, as the contact position shifts from the tip to the base of the sensor, the similarity initially increases before decreasing. This trend arises because the region near the tip extends beyond the camera's focal range, resulting in blurred contact areas in real tactile images. Conversely, in regions closer to the base, the proximity to the camera introduces significant lens distortion, leading to sparse particle distributions and increased interpolation errors in simulated tactile images.

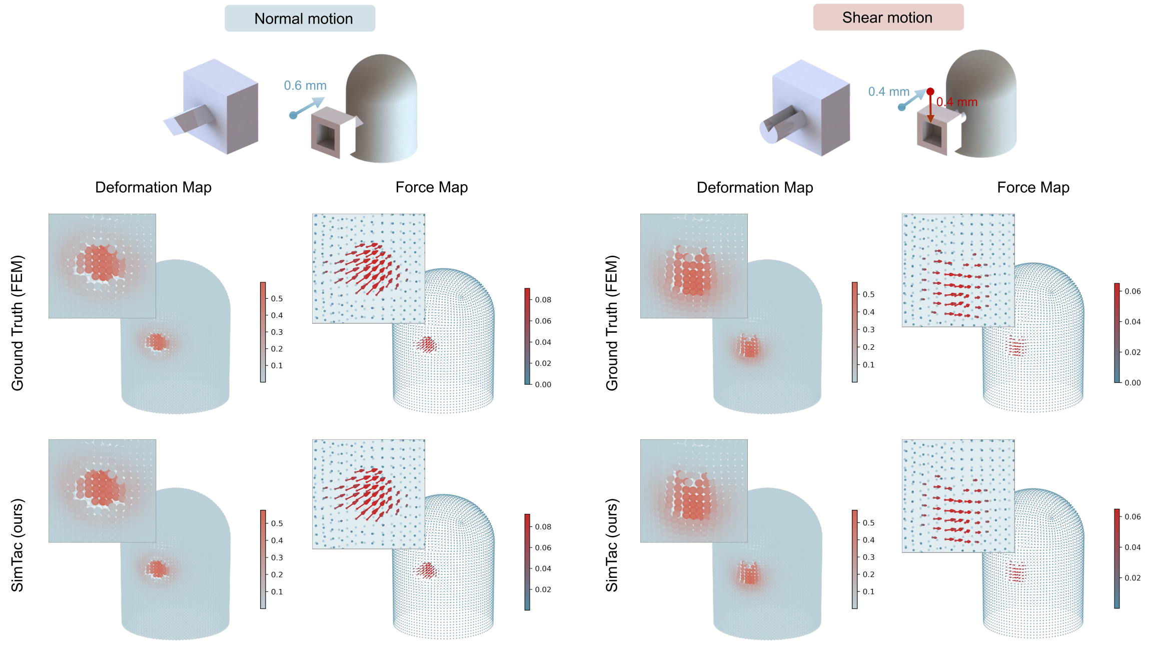

Figure 14: Evaluation of mechanical response simulation on the test set. The model was trained on data collected from 10 seen objects and evaluated on 4 unseen objects to assess its generalisation capability. 3D heatmaps were employed to visualise the distributions of deformation and force fields, where the colour represents the magnitude of the field data. The comparison shows that the simulated deformation and force values from the proposed model exhibit particle-level consistency with the ground truth computed from FEM, demonstrating the model's accuracy in simulating deformation and force when the sensor interacts with objects of various shapes.

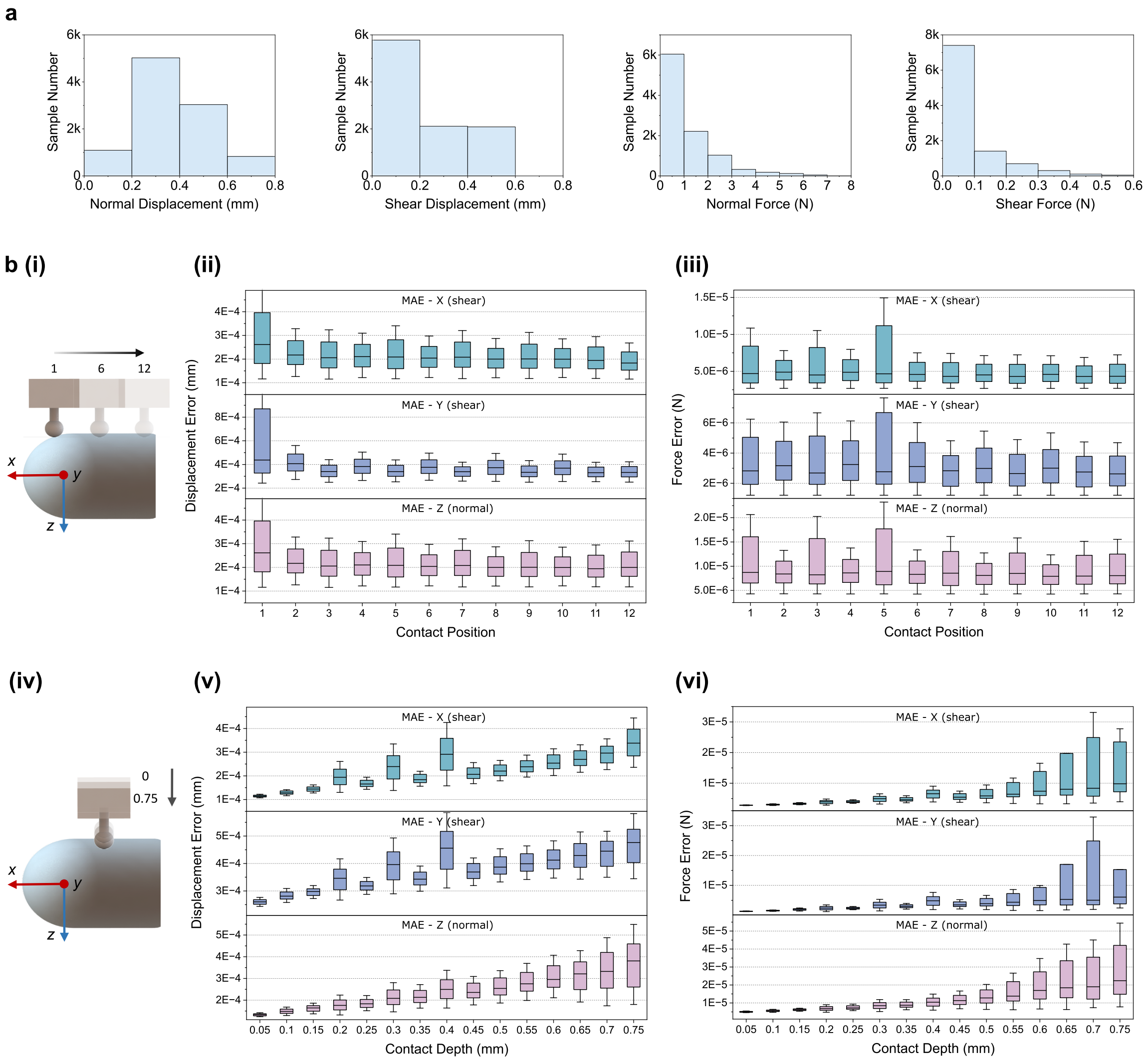

Figure 15: Evaluation of mechanical response simulation under different contact scenarios. **a**, Histogram of the tactile image quantity distribution across varying contact displacements and forces. **b**, Analysis of MAE between the simulated mechanical response and ground truth (computed by FEM) for the dense deformation and force fields at various (**i**)-(**iii**) contact positions and (**iv**)-(**vi**) depths. The deformation and force prediction errors are notably larger in contact region 1, which represents the transition zone between the sensor tip and its cylindrical body. This region experiences insufficient contact between the object and the sensor, resulting in substantial prediction errors. Additionally, the prediction errors increase with compression depth, as both displacement and total force values rise, thereby amplifying the overall prediction inaccuracies.

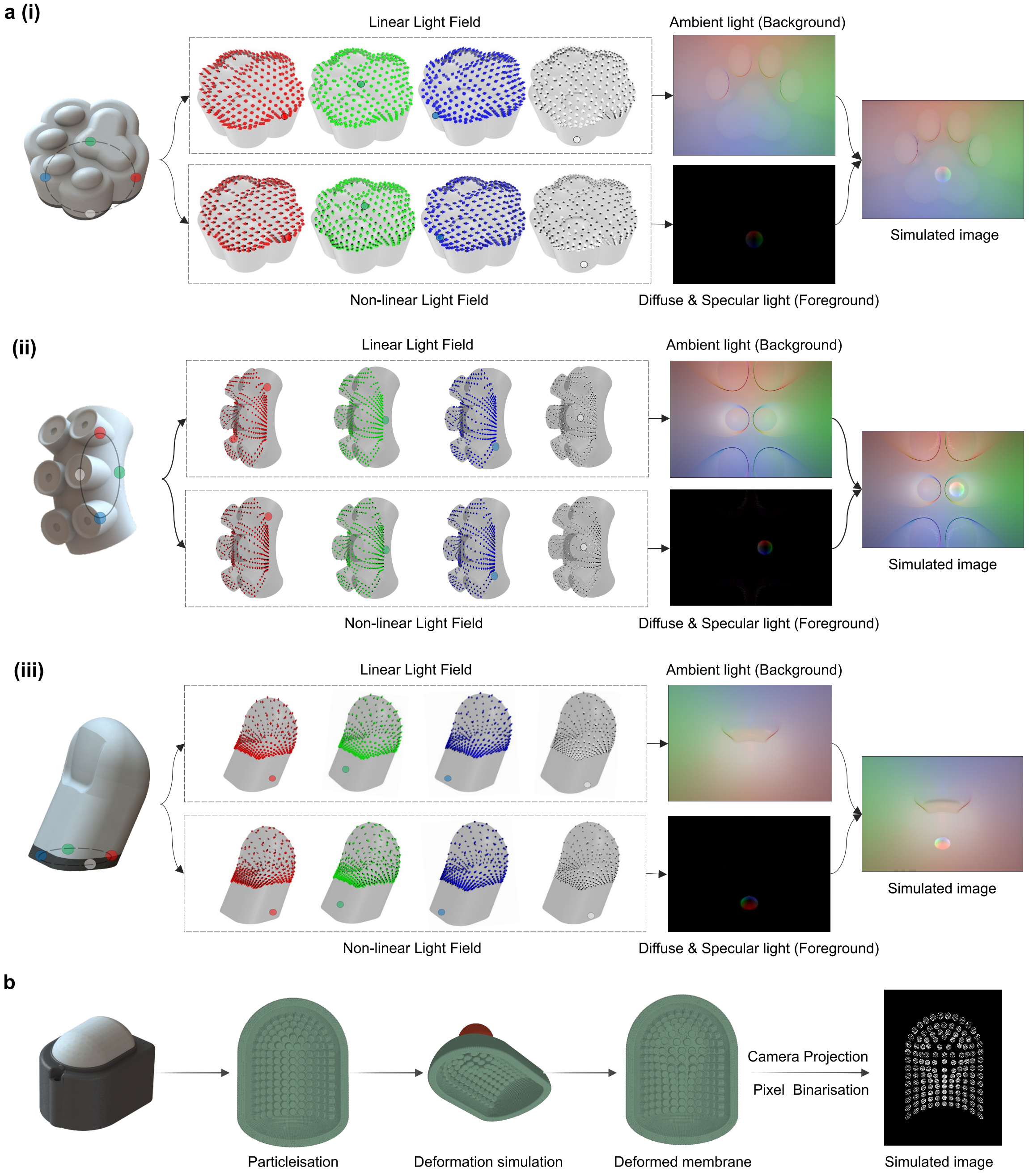

Figure 16: Simulation process for optical responses of biomorphic vision-based tactile sensors with diverse sensor shapes and optical configurations. **a**, Simulation process for optical responses of markerless GelSight-type tactile sensors: inspired from (**i**) cat paws, (**ii**) octopus tentacles, and (**iii**) human thumb. We reduced the density of the light field to facilitate visualisation and understanding. The tactile image is composed of a background image calculated from a linear light field and a foreground image calculated from a nonlinear light field. **b**, Simulation process for optical responses of marker-based TacTip-type tactile sensors: DigiTac sensor [14]. The simulator can replicate the deformation and motion of TacTip's physical pins during contact. The marker-based tactile images are generated by tracking the positions of particles within the tip region of the pins.

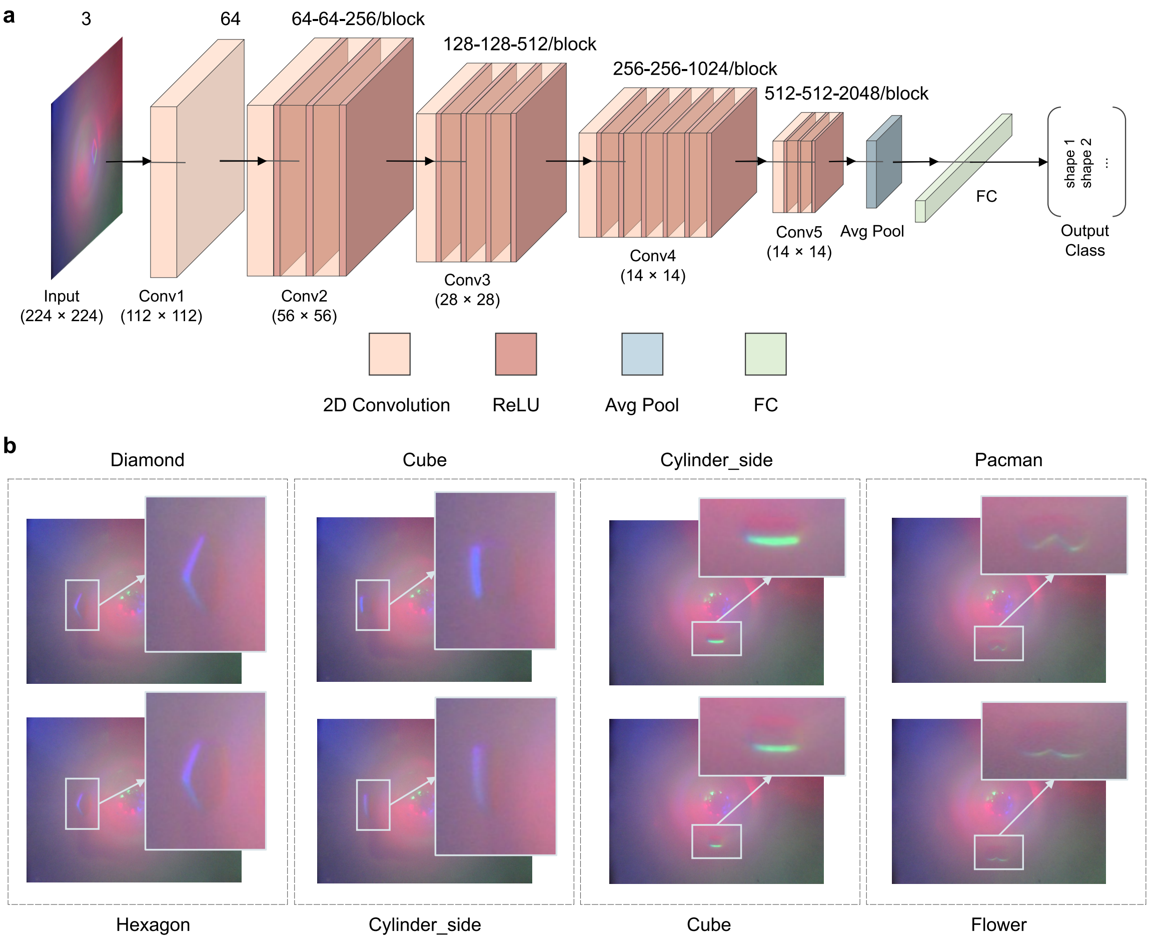

Figure 17: Network structure in Sim2Real object classification task and similar shapes leading to incorrect predictions. **a**, The ResNet50 [6] architecture is employed as the backbone for the proposed classification model. **b**, Example of similar shapes leading to incorrect predictions.

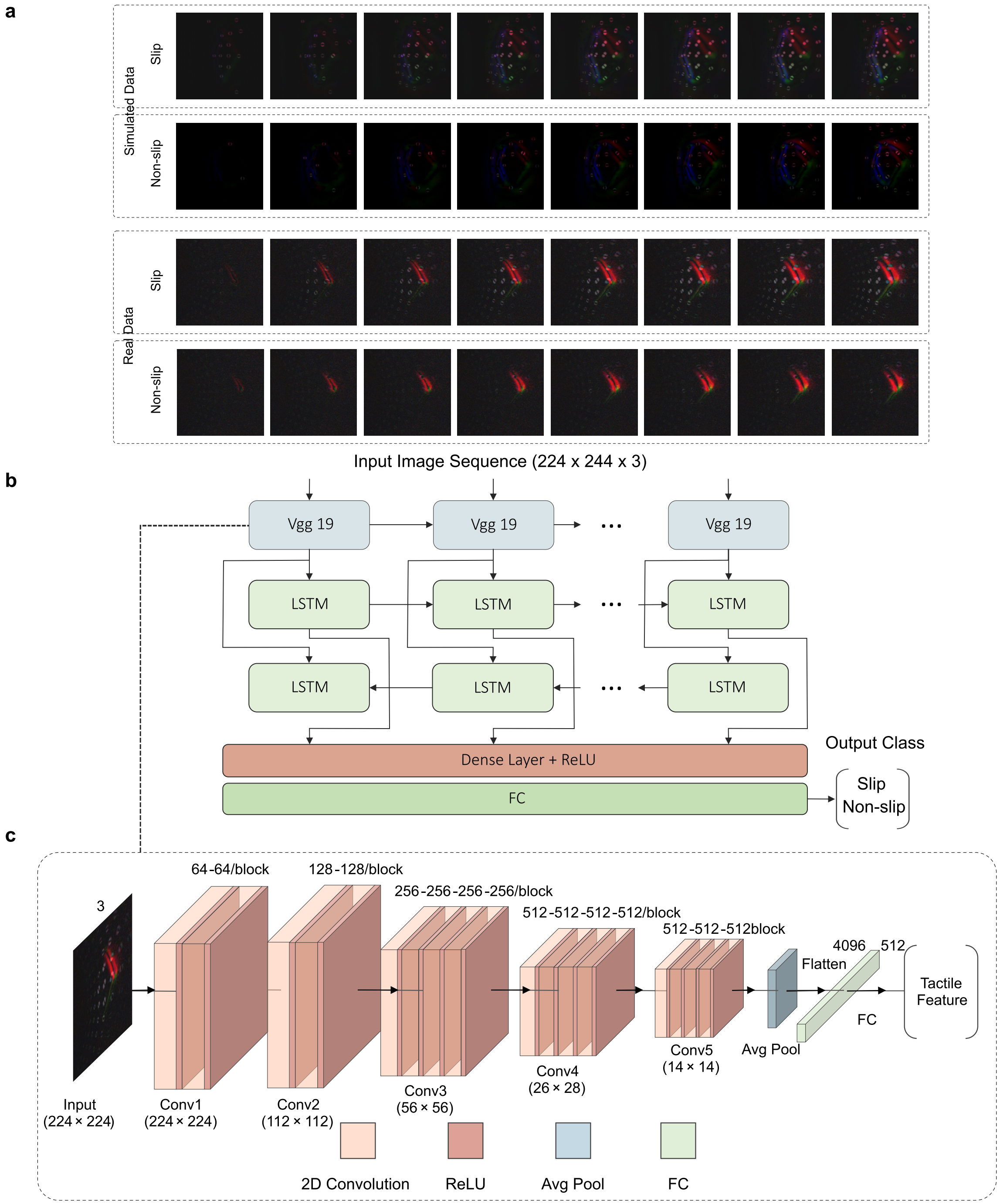

Figure 18: Network structure in Sim2Real slip detection task. **a**, Examples of simulated and real-world tactile image sequences as input to the network. In non-slip cases, the object's contour and surface markers move synchronously, while in slip cases, the object continues to move as the markers remain nearly stationary, indicating relative slip motion. **b**, VGG-19 [8] architecture is employed for tactile feature extraction, and LSTM model [9] is utilized for slip and non-slip prediction. **c**, Details of the employed VGG-19 architecture.

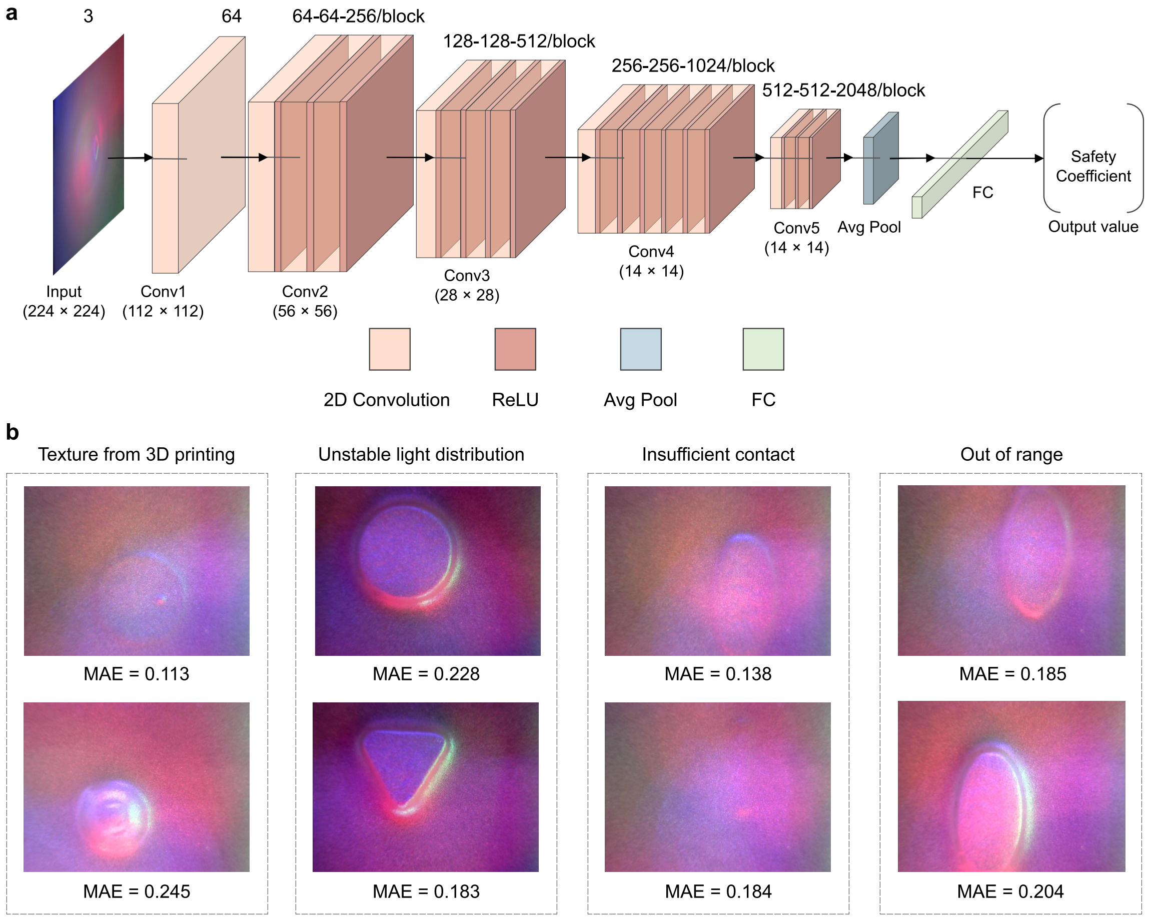

Figure 19: Network structure in Sim2Real contact safety assessment task and failure cases with large prediction errors. **a**, The ResNet50 [6] architecture is employed as the backbone for the proposed regression model. **b**, Examples of failure cases with large prediction errors in the safety coefficient. The higher Sim2Real error compared to Sim2Sim can be attributed to several factors: Firstly, 3D-printed objects exhibit surface textures absent in simulations, introducing artefacts that affect prediction error. Secondly, as the indentation depth increases, the deformation of the silicone surface alters light distribution, causing the overall image to darken. Thirdly, some real-world data suffer from insufficient indentation, resulting in tactile images that only capture partial contact contours rather than the full contact area. Finally, certain contact poses may cause objects to extend beyond the camera's field of view, limiting the completeness of tactile information.

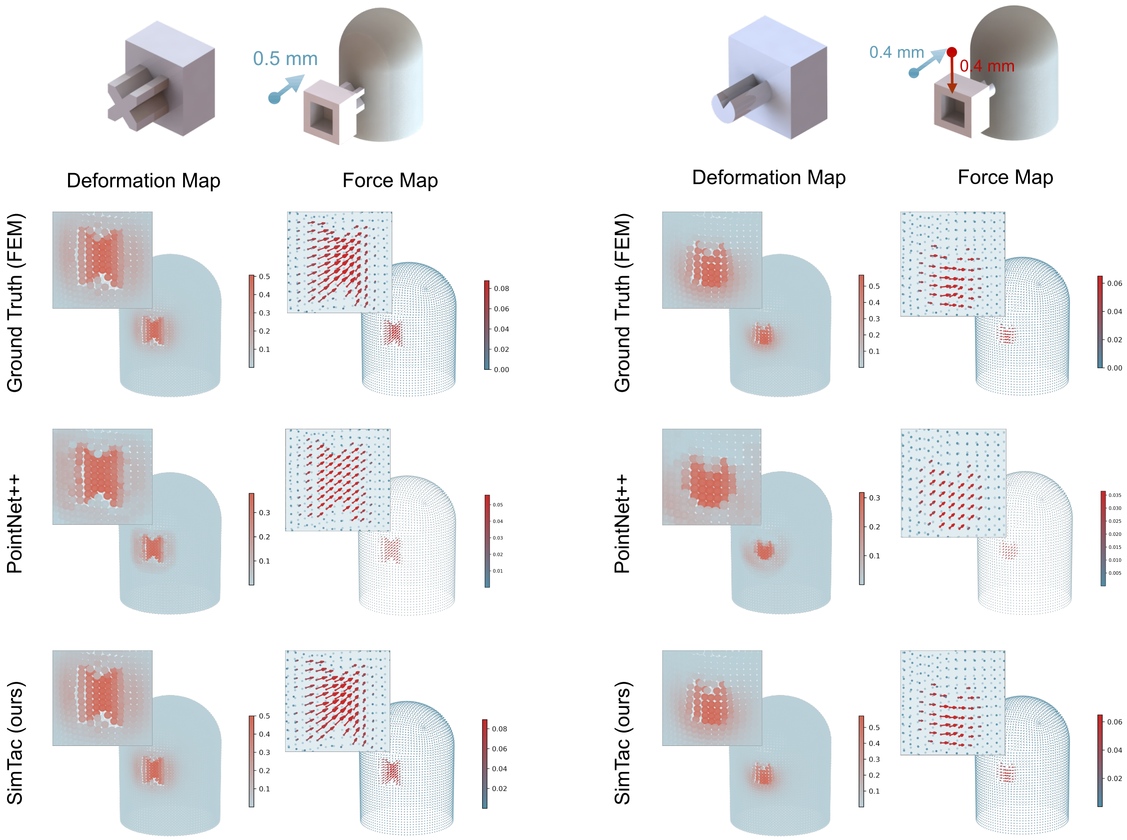

Figure 20: Visual Comparison of Deformation and Force Map Predictions between PointNet++ and SimTac.

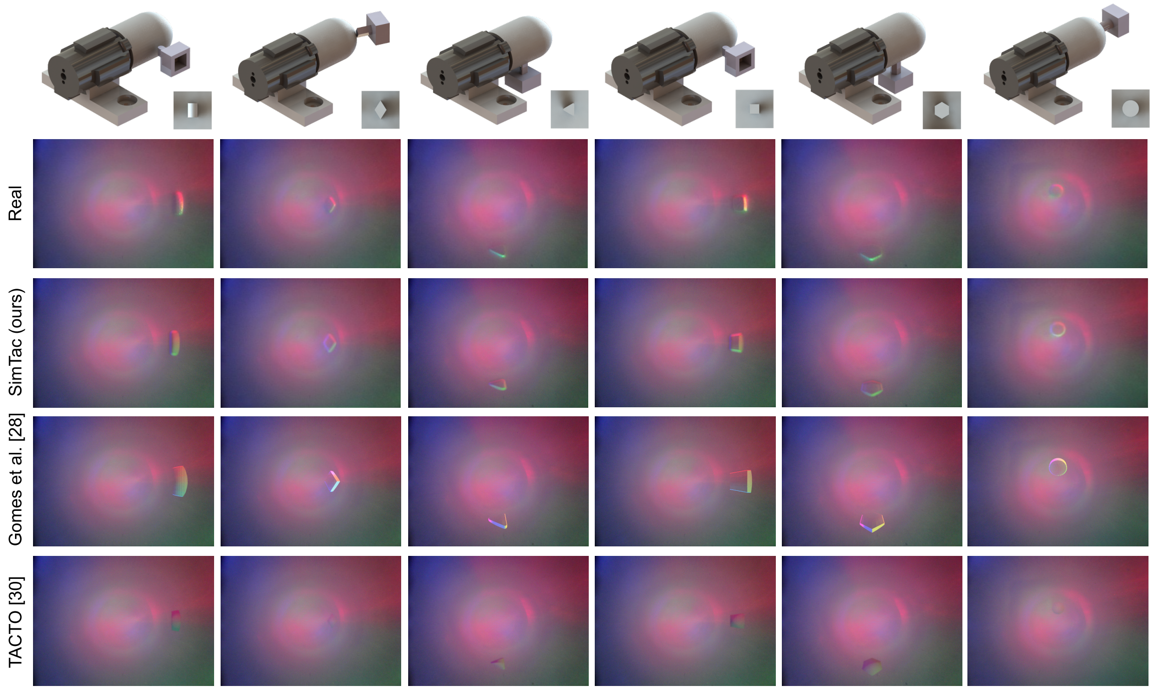

Figure 21: Optical response evaluation compared to advanced tactile simulators [11, 12]. SimTac produces smoother contact deformations than the baselines, thanks to its physics-based model that captures the silicone layer's physical behavior and avoids the sharp edges that typically result from model penetration method. It also generates more realistic color distributions through linear and nonlinear light-field rendering, which accounts for light propagation within the curved membrane rather than assuming purely linear transport.

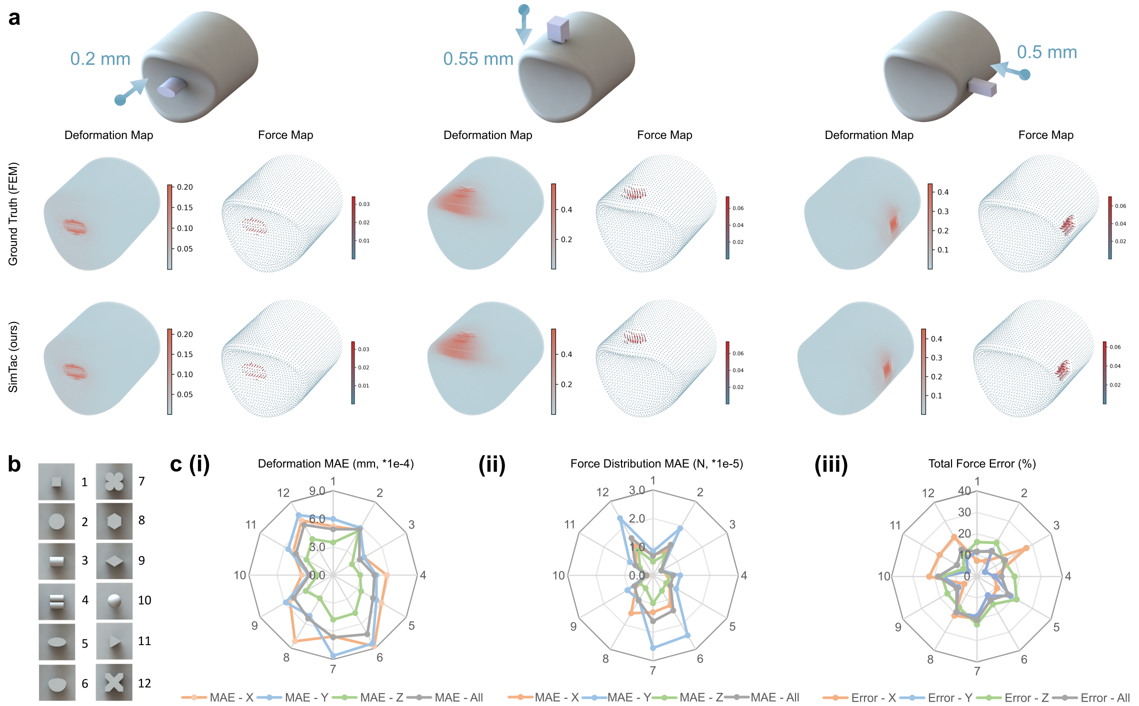

Figure 22: Evaluation of mechanical response simulation on an elephant-trunk-shaped sensor using the fine-tuned model. **a**, Comparison of 3D deformation and force maps between SimTac simulation and ground truth from FEM across different contact scenarios. **b**, Different indenters. **c**, The quantitative analysis results of the mechanical response simulation when in contact with different indenters. (**i**) MAE of deformation maps; (**ii**) MAE of force maps; (**iii**) Percentage error of the total force.

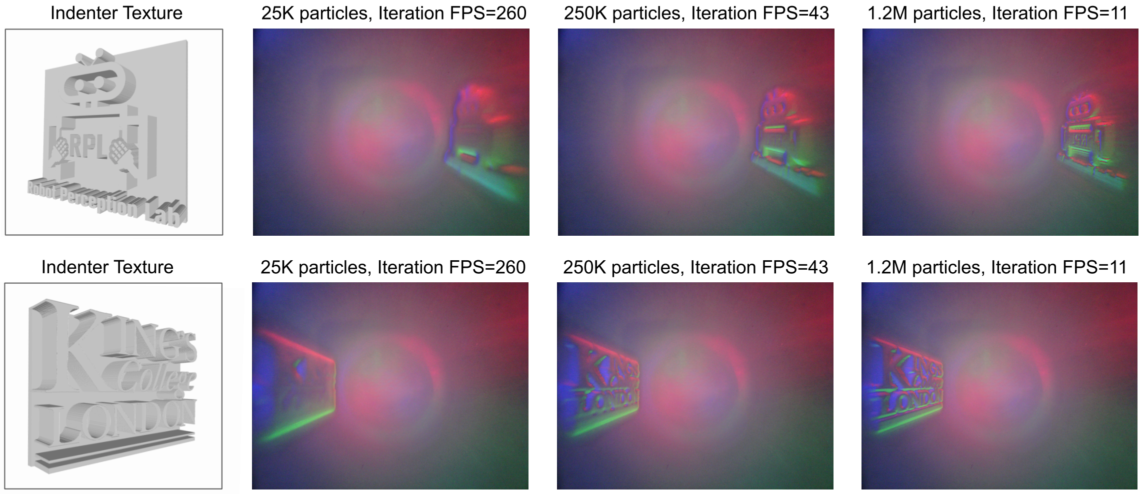

Figure 23: Simulation of fine surface textures using different particle resolutions.

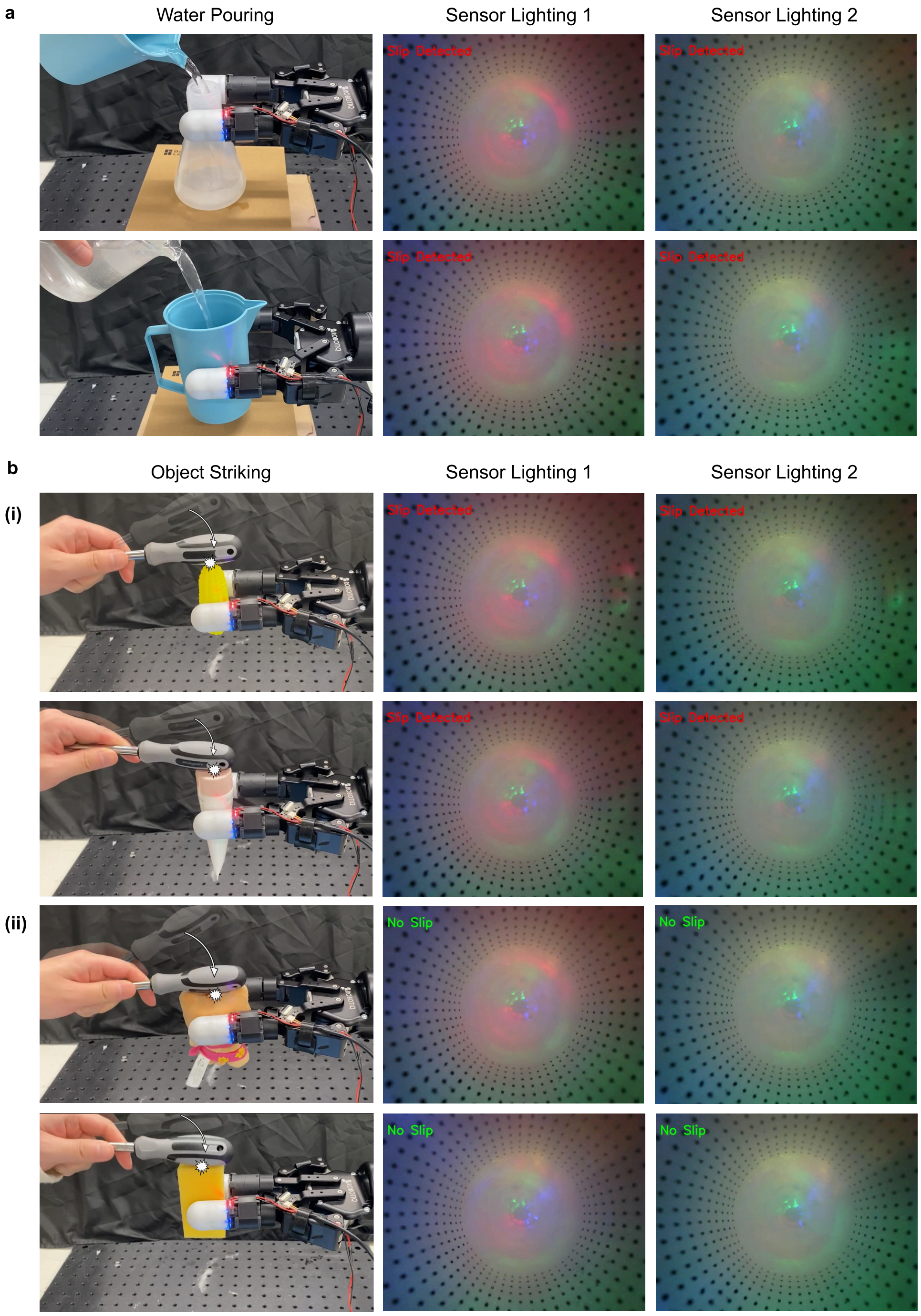

Figure 24: Demonstration of slip detection in **a**, water pouring and **b**, object striking tasks under different sensor lighting conditions.(**i**) Success cases in object striking task due to distinct contact geometric features; (**ii**) Failure cases in object striking task due to extreme softness of the tested objects.

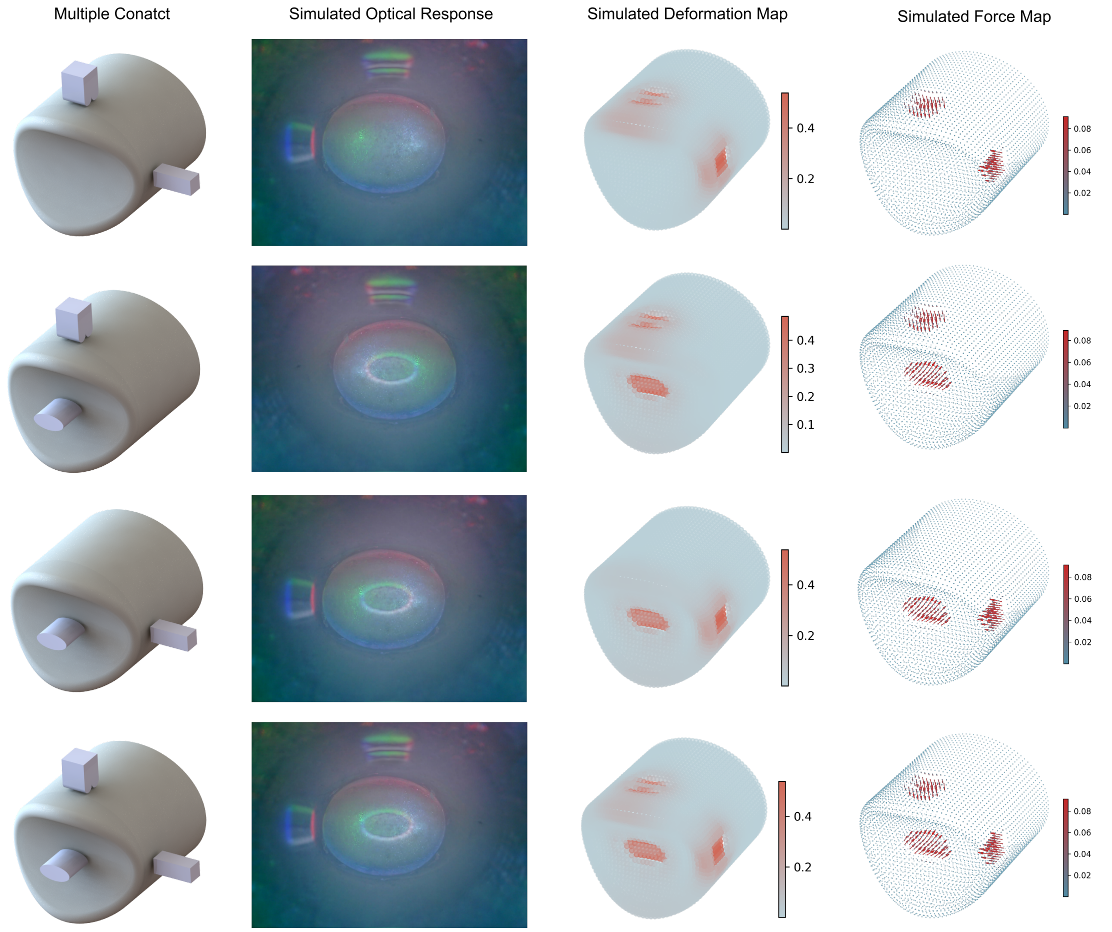

Figure 25: Simulation results of optical and mechanical responses to simultaneous multi-point contacts on an elephant-trunk-shaped sensor.
