# Supplementary figures and images for: SimTac: A Physics-Based Simulator for Vision-Based Tactile Sensing with Biomorphic Structures
Source: Cyborg Bionic Syst. 2026 Feb 24;7:0510. doi: 10.34133/cbsystems.0510 (PMC12929814; doi:10.34133/cbsystems.0510)

**a**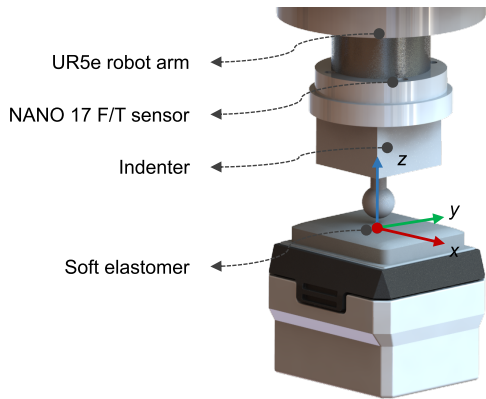

Real-world setup

**b**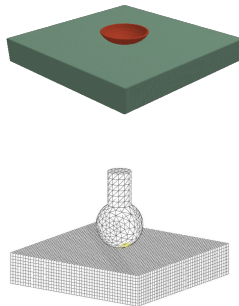

SimTac and FEM setup

**c**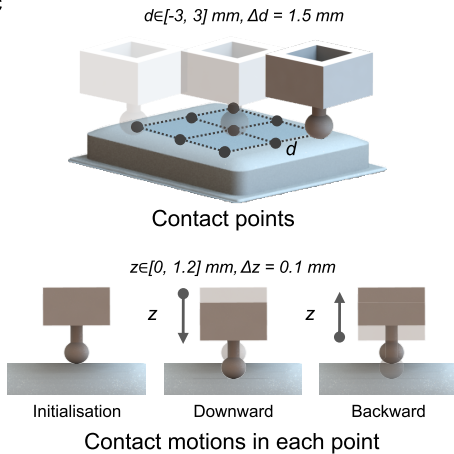

Supplement: Supplementary 1 — Supplementary Notes Tables S1 to S6 Figs. S12 to S25 Movies S1 to S6 [file cbsystems.0510.f1.zip › Figure 12.pdf]

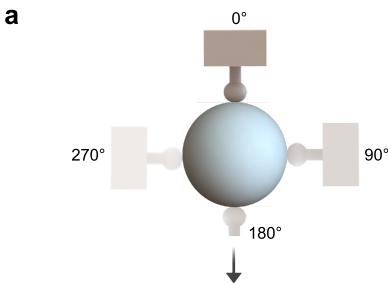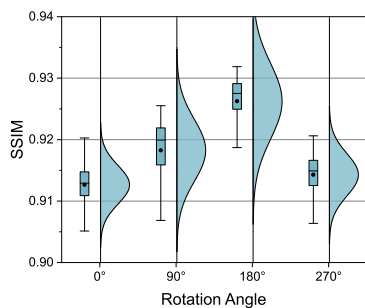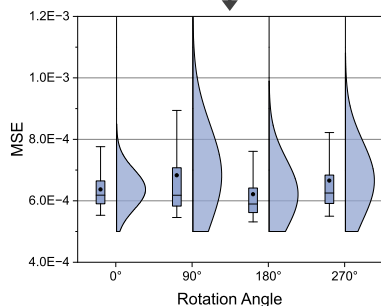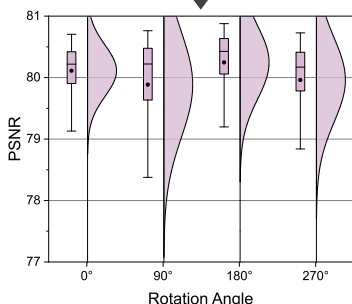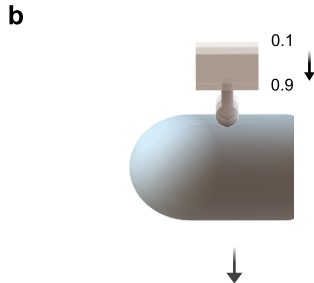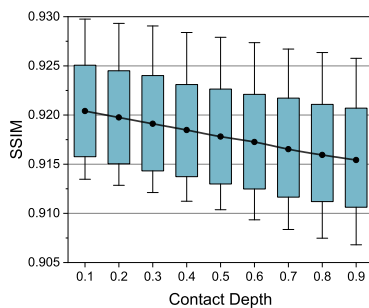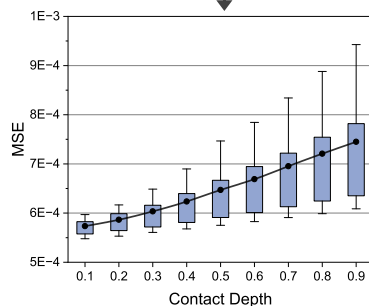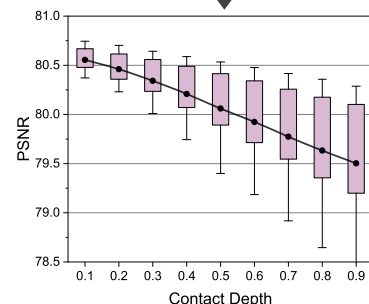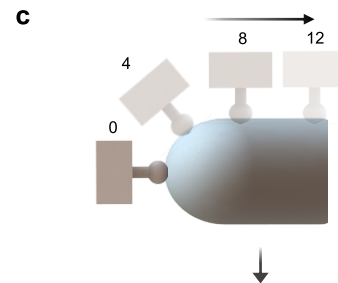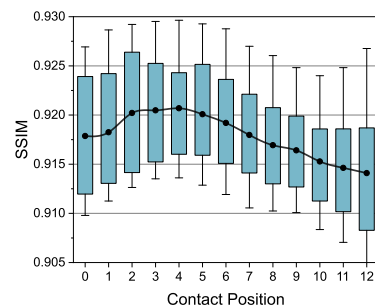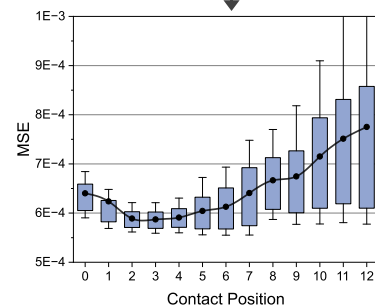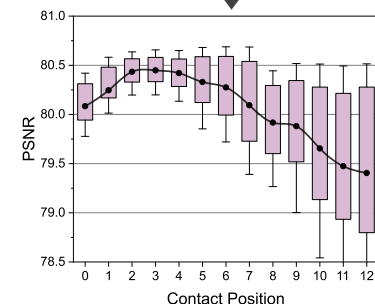

Supplement: Supplementary 1 — Supplementary Notes Tables S1 to S6 Figs. S12 to S25 Movies S1 to S6 [file cbsystems.0510.f1.zip › Figure 13.pdf]

**a**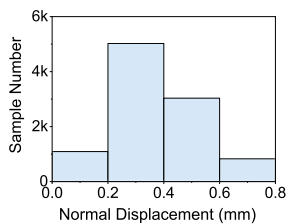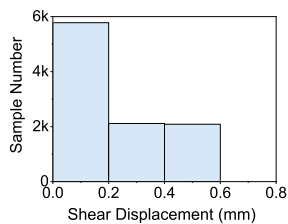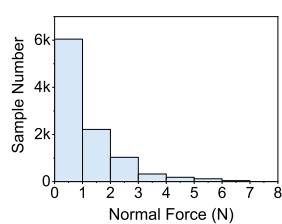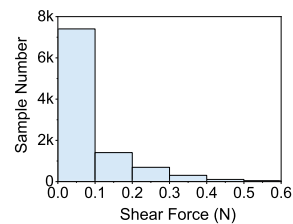**b (i)**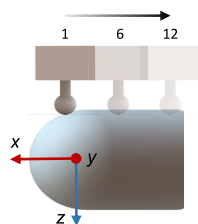**(ii)**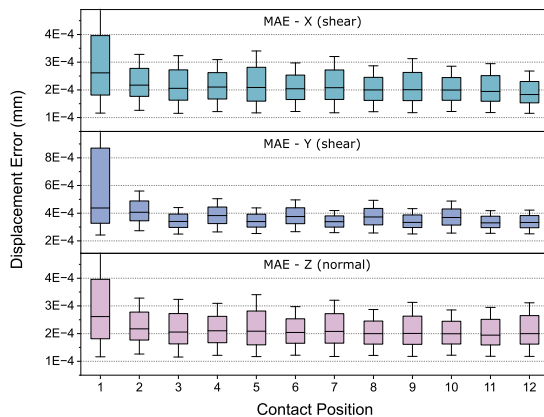**(iii)**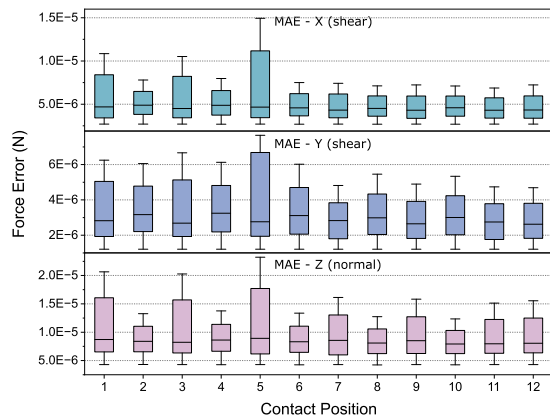**(iv)**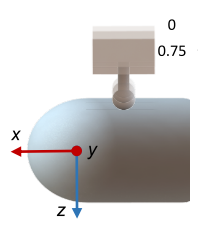**(v)**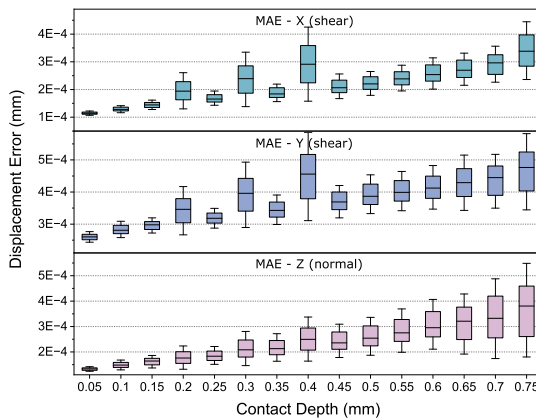**(vi)**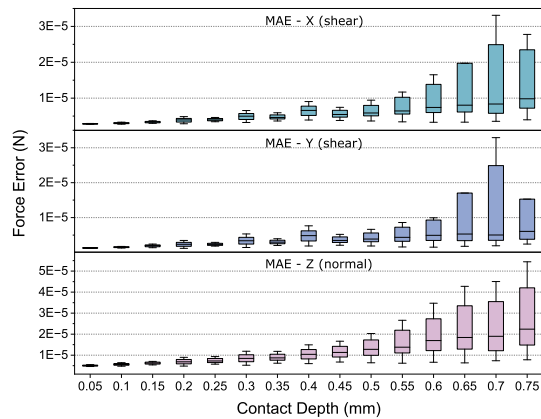

Supplement: Supplementary 1 — Supplementary Notes Tables S1 to S6 Figs. S12 to S25 Movies S1 to S6 [file cbsystems.0510.f1.zip › Figure 15.pdf]

**a (i)**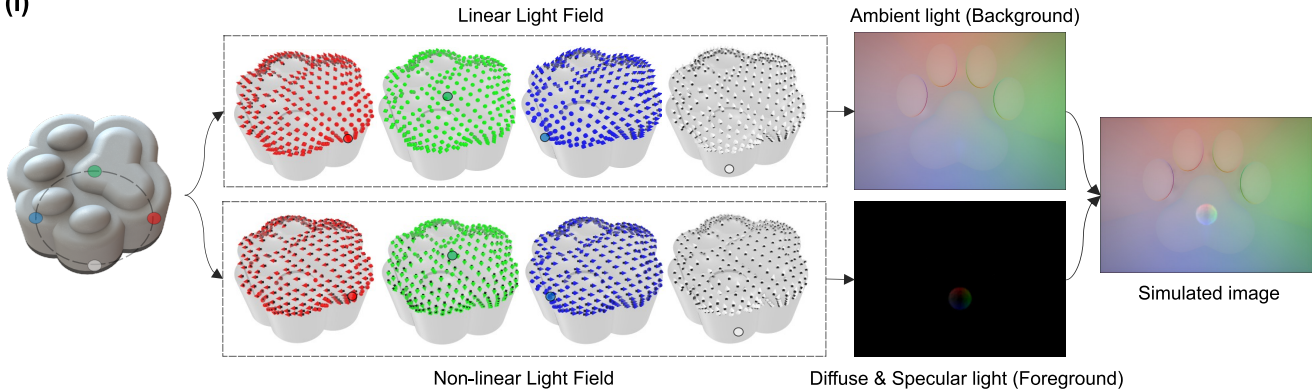**(ii)**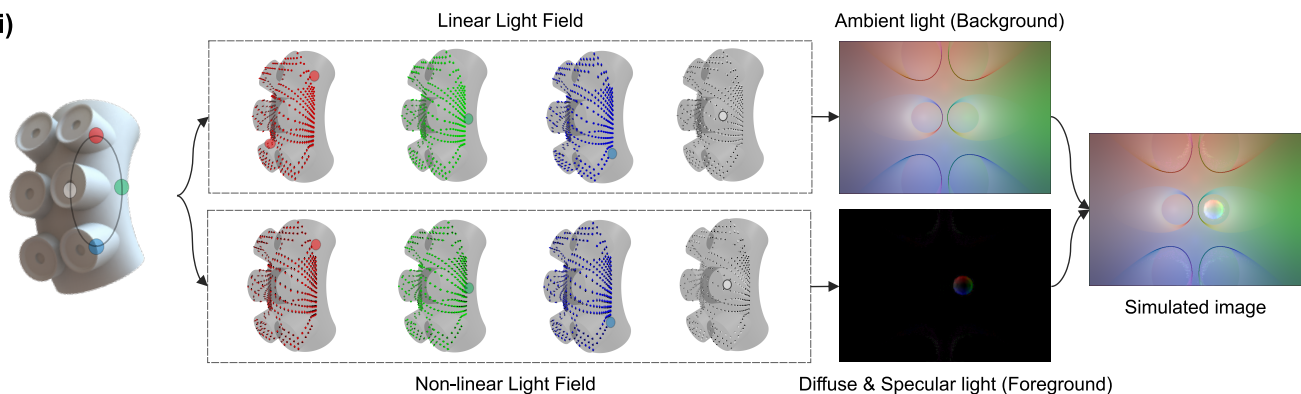**(iii)**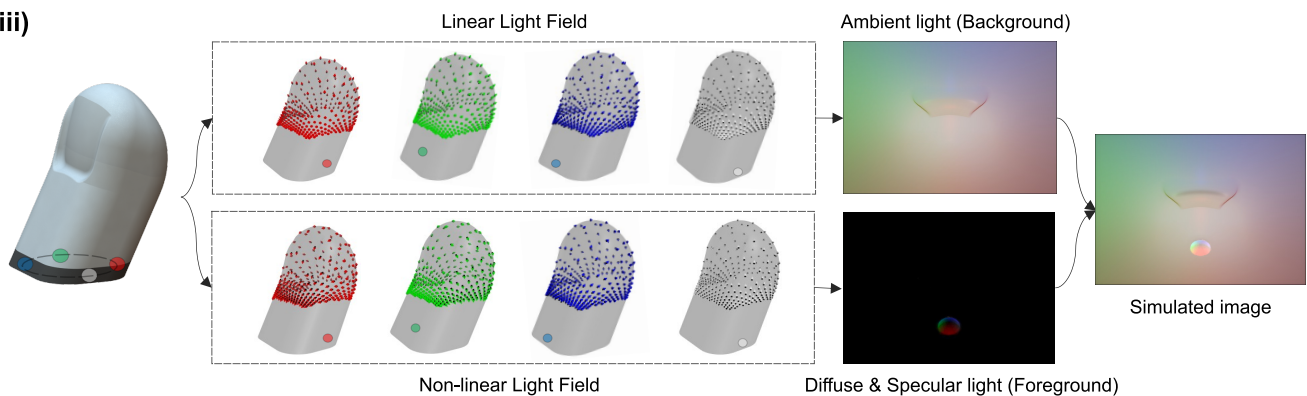**b**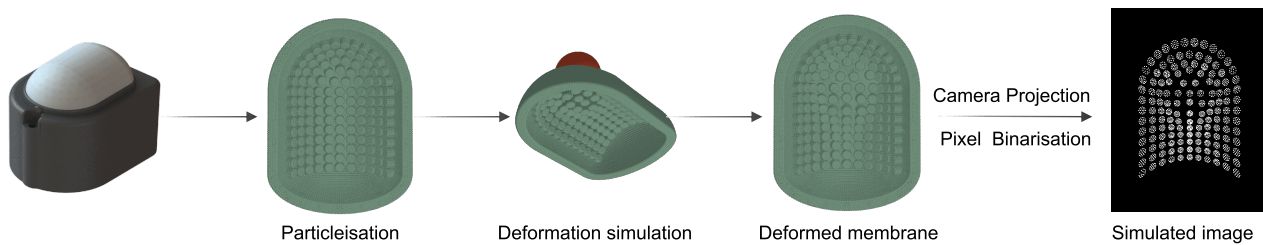

Supplement: Supplementary 1 — Supplementary Notes Tables S1 to S6 Figs. S12 to S25 Movies S1 to S6 [file cbsystems.0510.f1.zip › Figure 16.pdf]

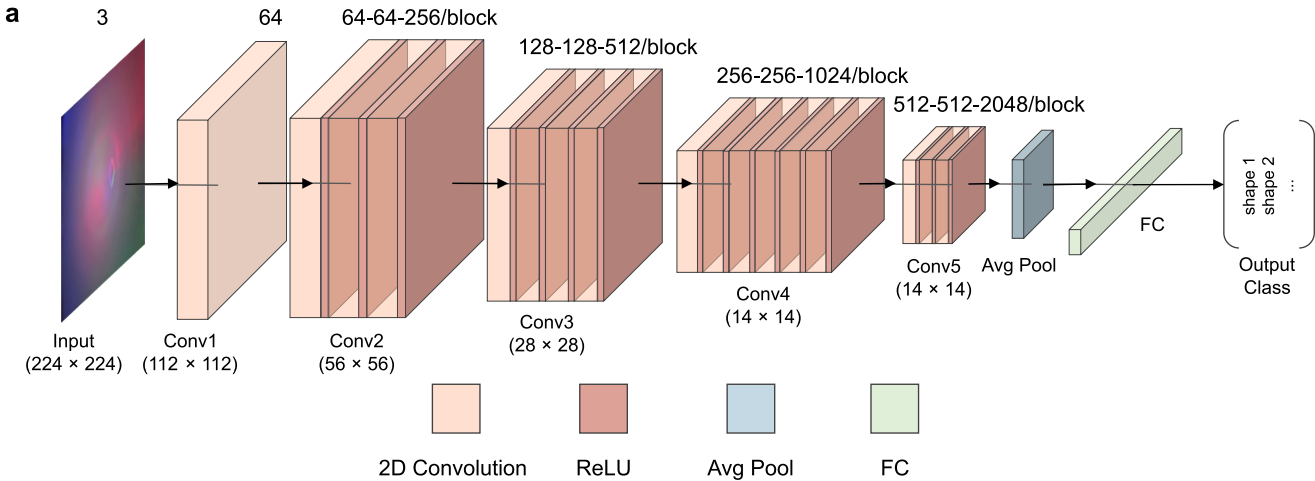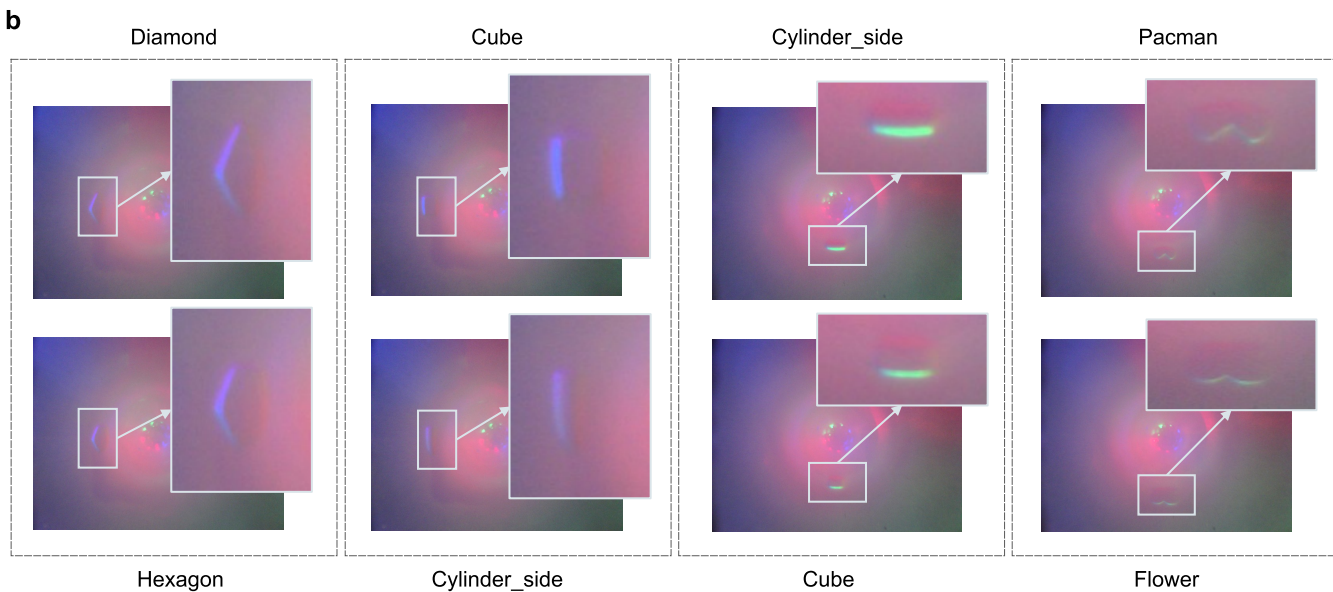

Supplement: Supplementary 1 — Supplementary Notes Tables S1 to S6 Figs. S12 to S25 Movies S1 to S6 [file cbsystems.0510.f1.zip › Figure 17.pdf]

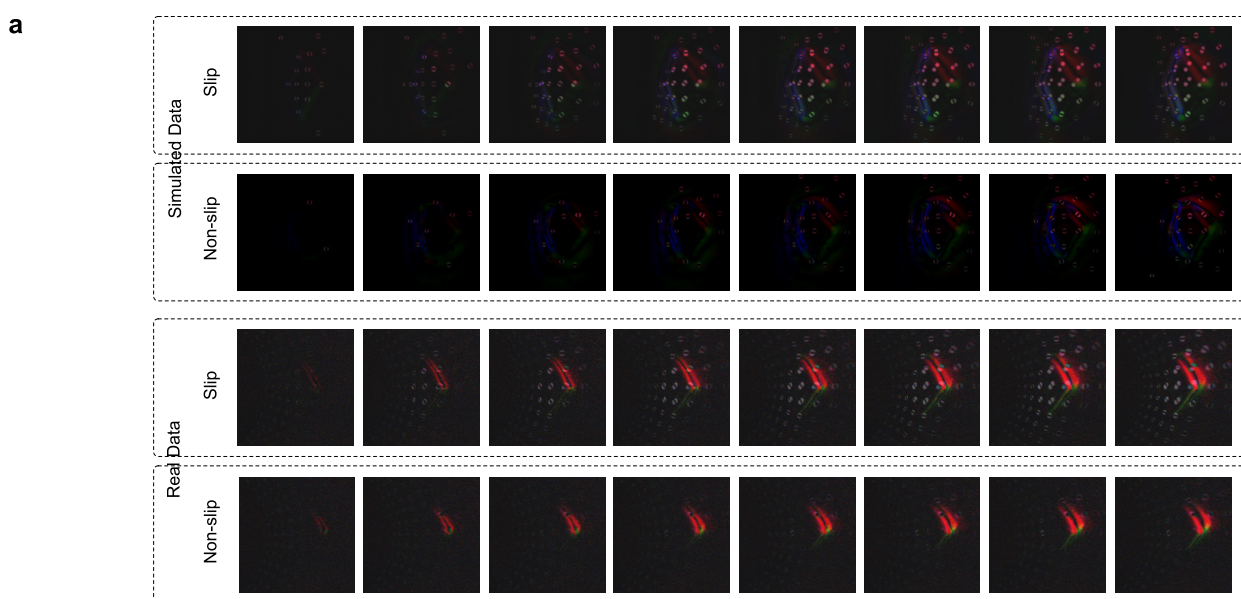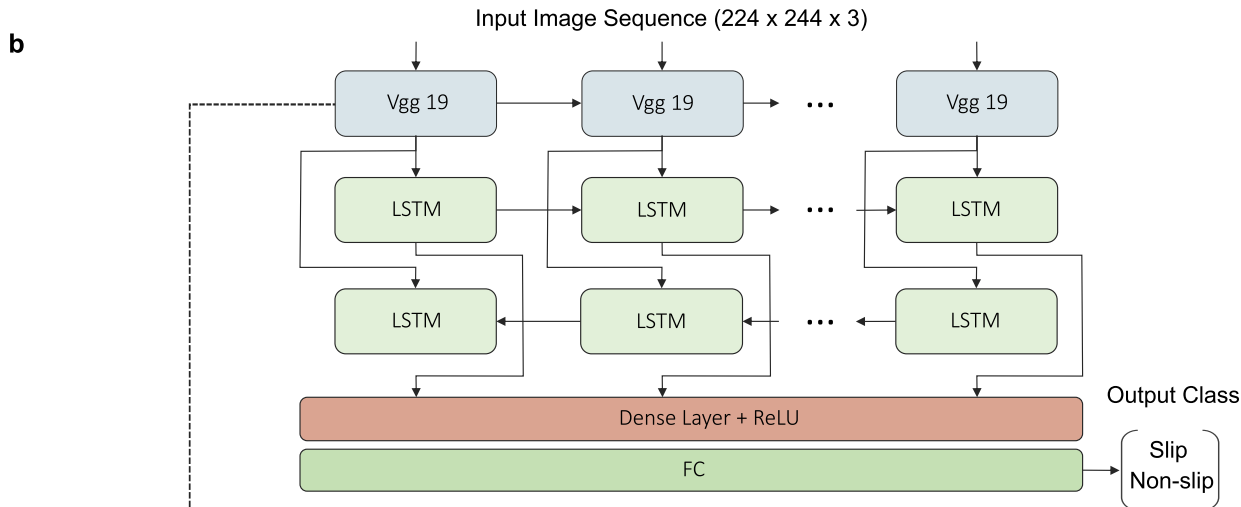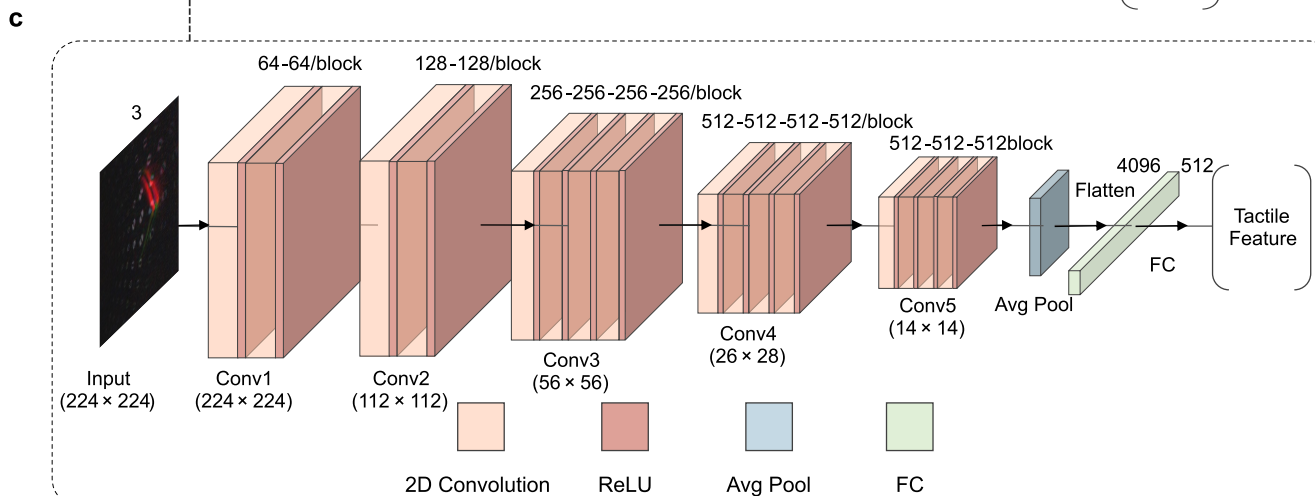

Supplement: Supplementary 1 — Supplementary Notes Tables S1 to S6 Figs. S12 to S25 Movies S1 to S6 [file cbsystems.0510.f1.zip › Figure 18.pdf]

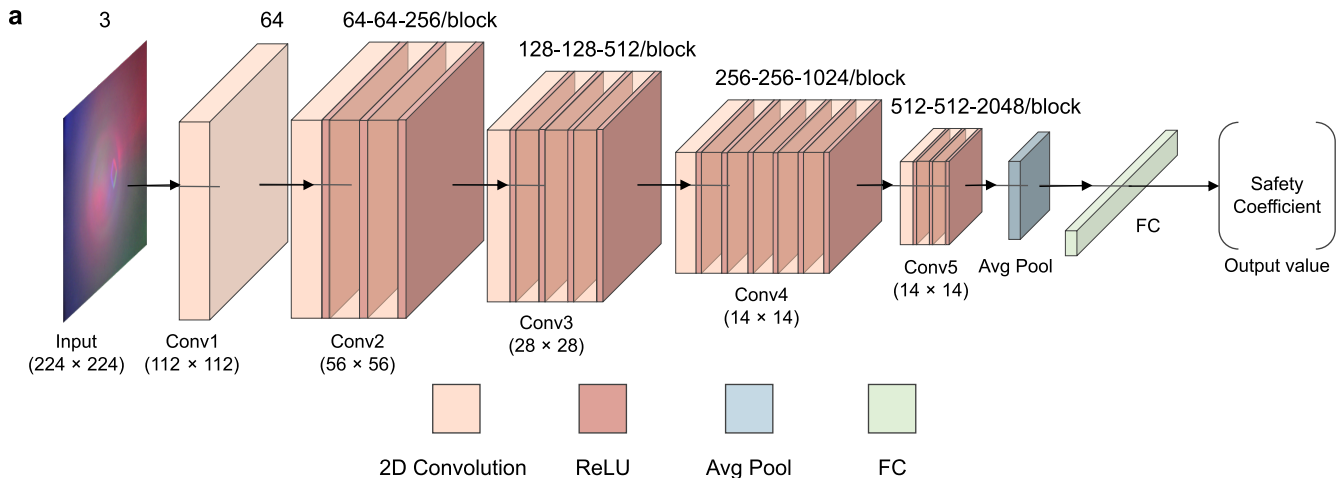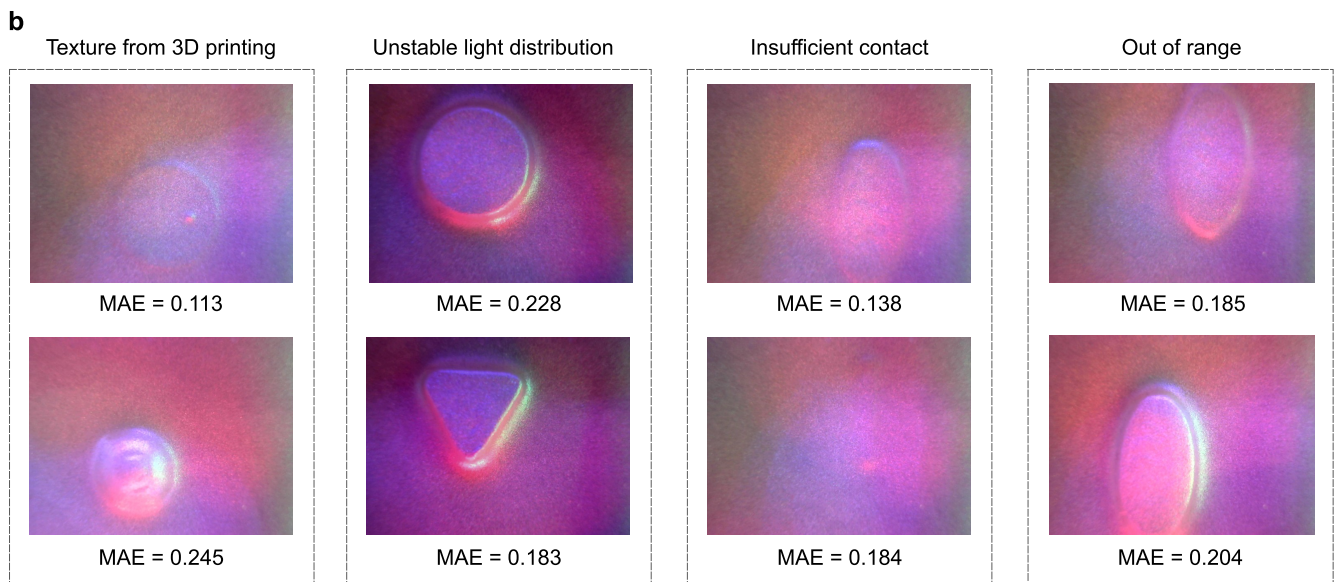

Supplement: Supplementary 1 — Supplementary Notes Tables S1 to S6 Figs. S12 to S25 Movies S1 to S6 [file cbsystems.0510.f1.zip › Figure 19.pdf]

Real

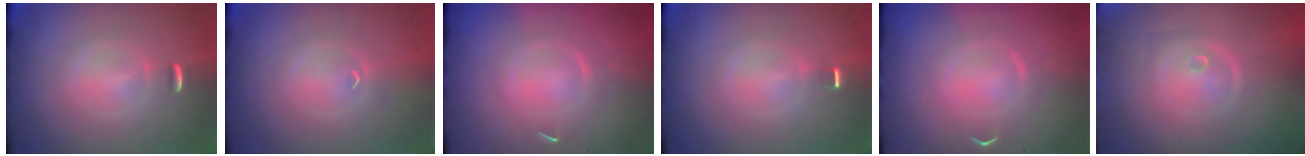

SimTac (ours)

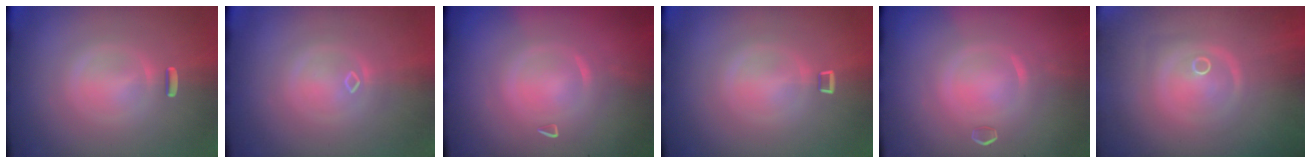

Gomes et al. [28]

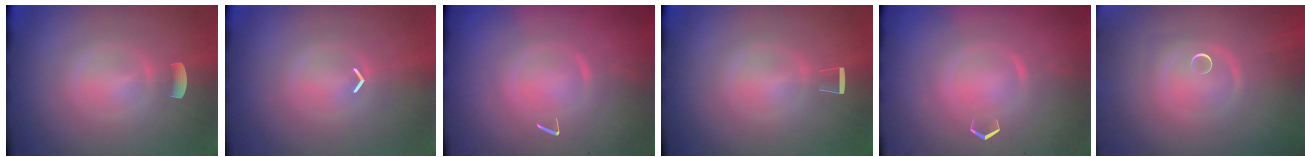

TACTO [30]

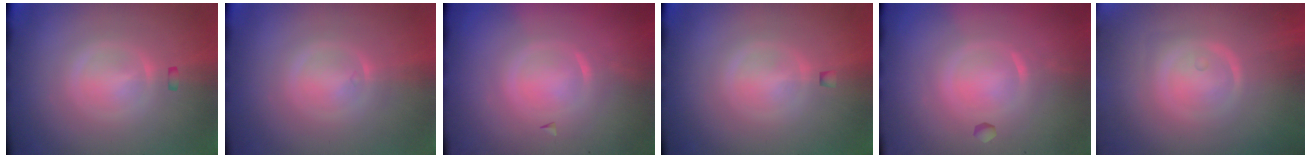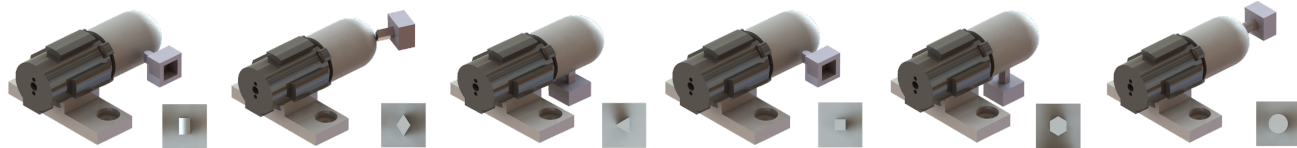

Supplement: Supplementary 1 — Supplementary Notes Tables S1 to S6 Figs. S12 to S25 Movies S1 to S6 [file cbsystems.0510.f1.zip › Figure 21.pdf]

Multiple Contact

Simulated Optical Response

Simulated Deformation Map

Simulated Force Map

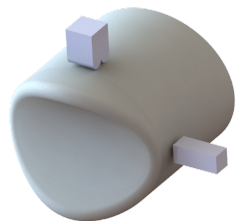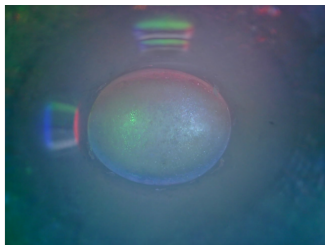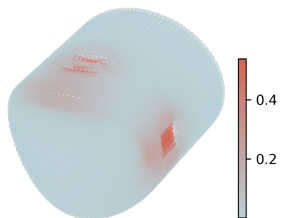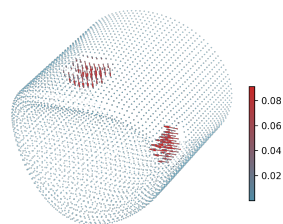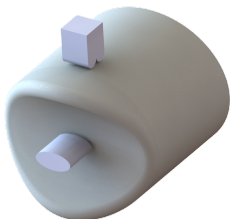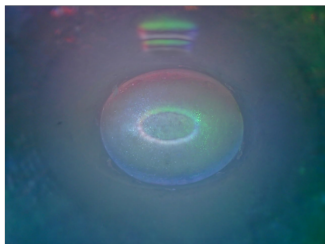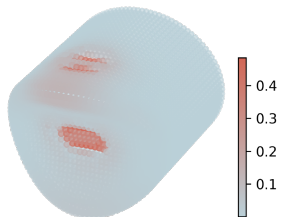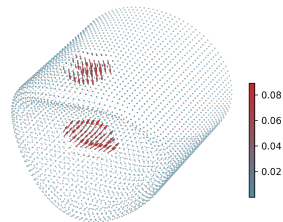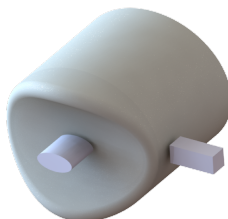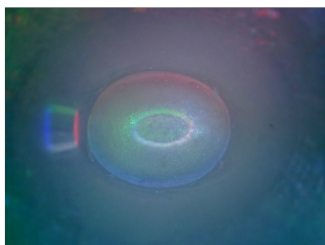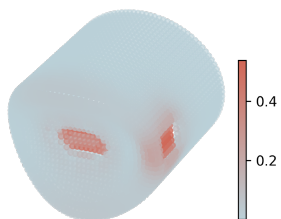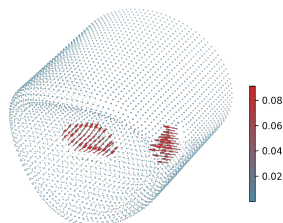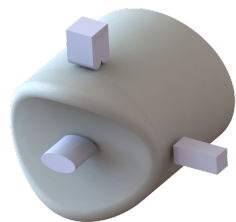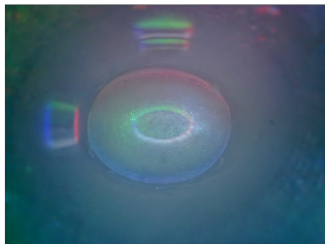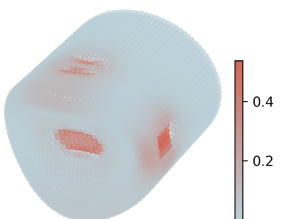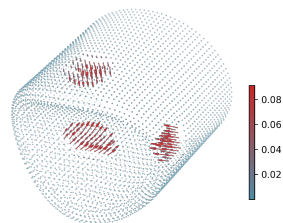

Supplement: Supplementary 1 — Supplementary Notes Tables S1 to S6 Figs. S12 to S25 Movies S1 to S6 [file cbsystems.0510.f1.zip › Figure 25.pdf]
